# Industrial Particulate Pollution and Historical Land Use Contribute Metals of Concern to Dust Deposited in Neighborhoods Along the Wasatch Front, UT, USA

**DOI:** 10.1029/2022GH000671

**Published:** 2022-11-01

**Authors:** Annie L. Putman, Daniel K. Jones, Molly A. Blakowski, Destry DiViesti, Scott A. Hynek, Diego P. Fernandez, Daniel Mendoza

**Affiliations:** ^1^ Utah Water Science Center U.S. Geological Survey West Valley City UT USA; ^2^ Department of Watershed Sciences Utah State University Logan UT USA; ^3^ Department of Geology and Geophysics University of Utah Salt Lake City UT USA; ^4^ Department of Atmospheric Sciences University of Utah Salt Lake City UT USA; ^5^ Department of City & Metropolitan Planning University of Utah Salt Lake City UT USA; ^6^ Division of Pulmonary Medicine University of Utah Salt Lake City UT USA

**Keywords:** dust, air pollution, Great Salt Lake, trace elements, strontium isotopes

## Abstract

The Salt Lake Valley, UT, USA, is proximal to the desiccating Great Salt Lake (GSL). Prior work has found that this lakebed/playa contributes metals‐laden dust to snow in the Wasatch and Uinta Mountains. Dust and industrial particulate pollution are also delivered to communities along the Wasatch Front, but their sources, compositions, and fluxes are poorly characterized. In this study, we analyzed the dust deposited in 18 passive samplers positioned near the GSL, in cities in and near the Salt Lake Valley for total dust flux, the <63 µm dust fraction, ^87^Sr/^86^Sr, and trace element geochemistry. We compared spatial patterns in metal flux and abundance with community‐level socioeconomic metrics. We observed the highest dust fluxes at sites near the GSL playa. Within the urban corridor, ^87^Sr/^86^Sr and trace element relative abundances suggest that most of the dust to which people are regularly exposed may be fugitive dust from local soil materials. The trace metal content of dust deposited along the Wasatch Front exceeded Environmental Protection Agency screening levels and exhibited enrichment relative to both the upper continental crust and the dust collected adjacent to GSL. Sources of metals to dust deposited along the Wasatch Front may include industrial activities like mining, oil refining, as well as past historical pesticide and herbicide applications. Arsenic and vanadium indicated a statistically significant positive correlation with income, whereas lead, thallium, and nickel exhibited higher concentrations in the least wealthy and least white neighborhoods.

## Key Points

- Dust near the Great Salt Lake playa differs from dust collected in nearby cities
- Metals in city dust can be traced to specific nearby industries
- Fugitive dust may be an important dust source for human exposure within cities

## 1 Introduction

Dust storms occur in arid regions globally, including the drylands of the southwestern United States. Breathing dust exacerbates cardiovascular distresses like asthma (Watanabe et al., 2011) and pneumonia (Cheng et al., 2008), and mortality in vulnerable populations (Mallone et al., 2011; Perez et al., 2008). In the North American southwest, dust fluxes have increased since the late 1800s due to agriculture, grazing, and mining (Brahney et al., 2013; Neff et al., 2008; Reynolds et al., 2010), and over the past 40 years, dust storm frequency has also increased, owing to land use intensification (Duniway et al., 2019), aridification due to consumptive water use (Wurtsbaugh et al., 2017), and drought‐inducing climate change (Tong et al., 2017; Williams et al., 2022). Given projected climate change and continued drought, dust emissions are projected to grow (Achakulwisut et al., 2019).

The rapidly growing cities at the foot of the Wasatch Mountains, UT (herein referred to as the Wasatch Front, in this study composed of Ogden, Bountiful, Salt Lake City, and Lehi (Canham & Semerad, 2021)) were home to over 2 million people in 2019 (United States Census Bureau, 2020). Concentrations of particulate matter <10 μm (PM_10_) in these cities routinely exceed national air quality standards (U.S. Environmental Protection Agency, 2022; Utah Department of Environmental Quality, 2019) owing in part to dust storms. The total dust flux is greatest for fronts with southerly winds (Steenburgh et al., 2012). The fine dry playa sediments of the shallow Great Salt Lake (GSL) to the northwest, the West Desert playa to the west, and Sevier Dry Lake playa to the south have been implicated as dust sources (Carling et al., 2020; Goodman et al., 2019; Skiles et al., 2018). Like other saline lakes globally, extractive water uses are shrinking the GSL (Doede & DeGuzman, 2020; Mallia et al., 2017; Wurtsbaugh et al., 2017). Local and national news coverage of the issue has highlighted that the expanding and increasingly weathered dry lakebed may increase fluxes of arsenic (As)—and other metal‐laden dust, further degrading already problematic air quality in the Central Wasatch (Flavelle, 2022; Kafanov, 2021; Spencer, 2021).

Enrichments of anthropogenically sourced trace elements (e.g., lead and arsenic) relative to average continental crust values have been observed at dust collection sites within and downwind of urban areas in Utah and Colorado (Goodman et al., 2019; Heindel et al., 2020; Reynolds et al., 2010). In some cases, this may point to effects of contaminated source sediments. However, in general, these findings suggest that small, high surface area geogenic mineral dust may scavenge gaseous or particulate pollution from mining, industrial, and mobile sources as the dust passes through the urban airshed (Marx et al., 2008). These sorbed metals tend to leach more readily in weak acids, meaning that they are more likely to be bioavailable (Dastrup et al., 2018; Goodman et al., 2019). If inhaled or ingested, these bioavailable metals may contribute to toxic exposure in humans and biota during and after dust events through inhalation and ingestion pathways, though that effect hasn't yet been quantified.

The effect of anthropogenic and industrial activities has been documented in the airsheds of populated areas near the Wasatch Front. In the Wasatch and Uinta Mountains, prior studies found that dust is a major source of trace elements, including heavy metals and rare earth elements (REEs), to snowpack and lake sediments (Carling et al., 2012; Dastrup et al., 2018; Goodman et al., 2019; Munroe et al., 2015; Reynolds et al., 2010). Analysis of lake sediment cores implicated mining and smelting activities. Along the Wasatch Front, the highest concentrations of trace and REEs are present downwind of the most populated and industrial Salt Lake Valley (Dastrup et al., 2018). Of these studies, only Goodman et al. (2019) explicitly investigated dust fluxes and geochemistry into urban areas along the Wasatch Front.

The interaction between dust originating on the GSL playa, other regional playas, and from other natural sources, with anthropogenic and industrial metals contributions is currently unconstrained in the Salt Lake Valley. However, following other air quality research, spatial variability within the Salt Lake Valley (e.g., Mendoza et al., 2019; Mitchell et al., 2018), and the dispersion of industrial emitters across the valley, we expect spatial variability in trace metals in the Salt Lake Valley, with specific metals abundances related to specific anthropogenic processes. We test this hypothesis using spatially distributed sampling, including dust samplers proximal to the GSL playa as well as throughout the greater Salt Lake Valley area from 2018 to 2019. We trace variability in geochemistry to specific processes and industrial emitters across the valley using spatial patterns of relative abundance and flux. Because other Salt Lake Valley pollution studies have identified differential exposure to pollution on the basis of racial, religious, and income demographics (Cobley et al., 2021; Collins & Grineski, 2019; Demetillo et al., 2021; Mendoza, 2020), we evaluate how the spatial variation in geochemistry aligns with sociodemographic characteristics. Finally, we delve into the sources of lanthanum (La), copper (Cu), thallium (Tl), molybdenum (Mo), and arsenic (As) as pollution. This analysis provides new information about how and why dust composition varies in a relatively small region and has implications for environmental health.

## 2 Methods

### 2.1 Study Area Background

#### 2.1.1 Topography, Hydrology, and Meteorology and Air Quality

**Figure 1:**
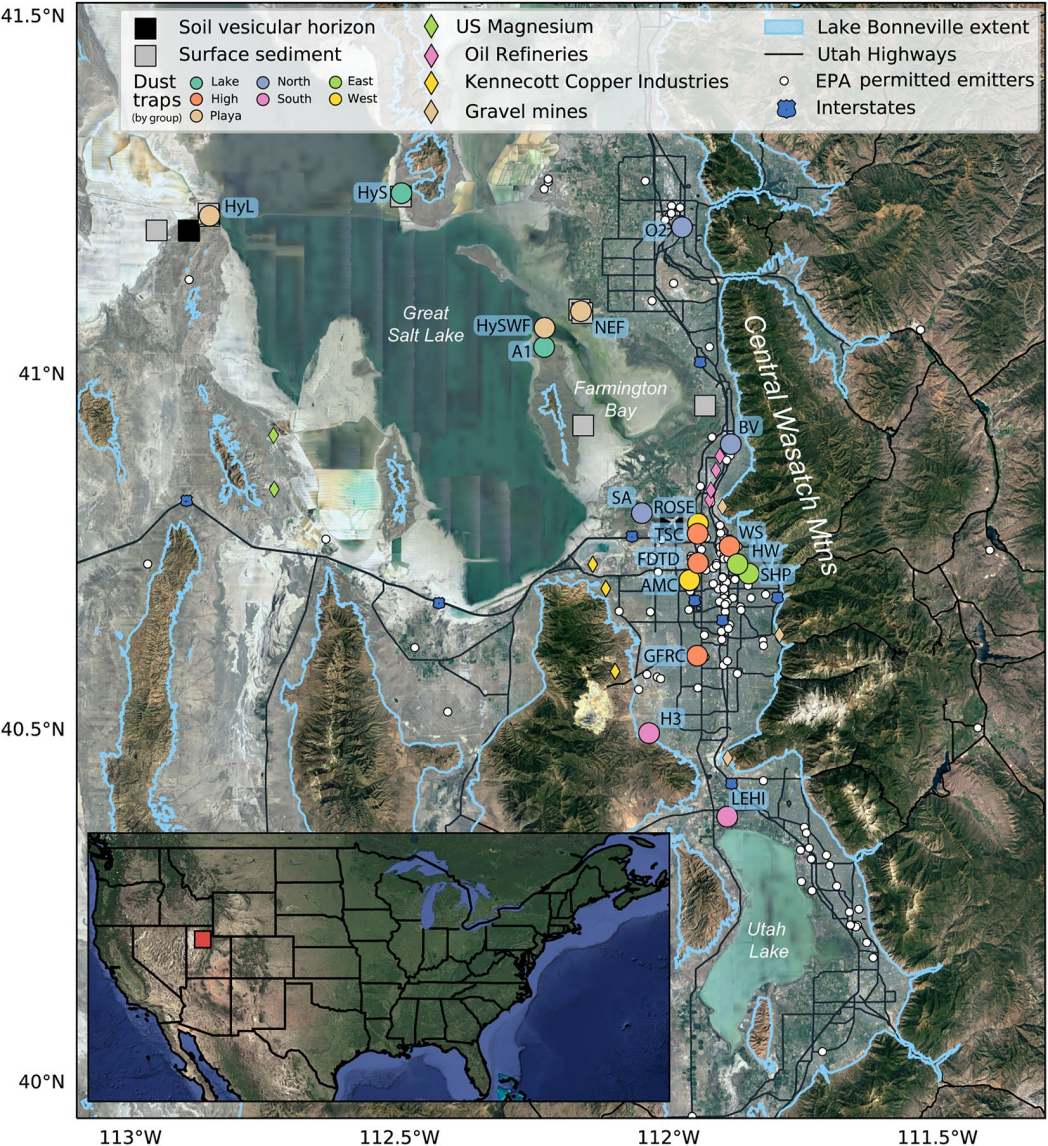
Map of the study region in northern Utah. Map shows sites of dust collection and surface sediment sampling.

The Salt Lake Valley is situated on the eastern end of the Basin and Range province, where semi‐arid valleys are interspersed with north‐south oriented mountain ranges. The Salt Lake Valley is constrained on the east and west by the Central Wasatch and the Oquirrh Mountains, respectively (Figure 1), and lies southeast of the hyper‐saline, endorheic GSL, the last remainder of paleo Lake Bonneville. Dessication from previous modern lake extents left behind expanses of fine‐grained salty playas. The primary water sources to the lake are the Jordan, Weber and Bear rivers (see Figure 10 in Baskin et al. (2002)), which drain to the lake from the east.

Wintertime weather is characterized by high pressure‐driven persistent cold air pools, which trap local pollution in a cold airmass in the valley (Gorski et al., 2015; Lareau et al., 2013). Summer season weather is hot and dry, with occasional convective precipitation and pollution characterized by high ozone (Horel et al., 2016) as well as periodic smoke from forest fires in the region. Dust events occur most often during fall and spring in association with synoptic high wind events from the north or northwest (Steenburgh et al., 2012) occurring in conjunction with sufficiently low soil moisture to allow for transport.

#### 2.1.2 Demographics, Land Uses, and Pollution Sources

The study area comprises Salt Lake, Davis, Tooele, and Utah Counties, with a combined population of more than 2 million people in 2019 (United States Census Bureau, 2020). The counties are largely white, with Tooele, Davis and Utah county estimating nearly 90% of their populations as non‐Hispanic White, and about 10% Hispanic or Latino of any race. Salt Lake County is somewhat more racially diverse, with 70.3% of the population estimated to be non‐Hispanic White, 2.2% Black, 1.4% Native American, 4.6% Asian, 1.8% Pacific Islander, and 2.9% from two or more races. 18.8% of the population are Hispanic or Latino of any race (United States Census Bureau, 2020). Within the Salt Lake Valley, the majority of non‐white population lives west of interstate highway I‐15, with the affluent east bench being largely white (Collins & Grineski, 2019). Both the racial and economic divides of Salt Lake County follow well documented racial and income divisions inherited from redlining practices of the 1940s (Nelson et al., 2021).

The study area comprises a variety of land uses. Most of the land area is high and moderate intensity urban, which includes private residences, roadways and commercial businesses. The study area also includes industrial and agricultural land uses. Natural land cover types surrounding the populated valley include rangeland, forests, wetlands where the rivers drain into the GSL, and the GSL playa.

The Central Wasatch, including the Salt Lake Valley, Bountiful, Ogden, and Lehi are home to a diversity of industrial activities. Situated on the far west side of the Salt Lake Valley is the Kennecott open pit porphyry copper mine and its refining and smelting operations (Figure 1). To the northwest there is a notable magnesium production facility that is also an Environmental Protection Agency (EPA) superfund site, munitions testing grounds, and a major international airport. To the north of the valley industries include oil refining, chroming, and refuse incineration. To the east and south, as well as throughout, there are large open pit gravel mines. Salt Lake City contains the crossroads of I‐80 and I‐15, two interstate highways, and automobile traffic is a large contributor to existing air quality issues (Long et al., 2002; Sells, 2019), such as particulate matter <2.5 μm (PM_2.5_) pollution events and high summertime ozone concentrations (U.S. Environmental Protection Agency, 2022).

Kennecott Utah Copper LLC runs the largest mining operation in the Salt Lake Valley, extracting and processing ore from the Bingham Canyon mine. The mine, a large Cu‐Mo‐Au deposit situated to the west of the Salt Lake Valley, contributes high Cu concentration particulate matter to lake sediments in the Uintas (Reynolds et al., 2010), and dust collected in the Wasatch and Uinta mountain ranges between 30 and 60 miles to the east (Goodman et al., 2019; Munroe, 2014). The mine also produces Mo as a secondary product (U.S. Geological Survey, 2021) and processing at the mine includes concentrating Mo. Fitzpayne et al. (2018) observed that this Cu‐Mo‐Au deposit also has high Tl abundances in rock samples compared to continental crust. As a result, this mining, smelting, and concentrating operation releases measureable amounts of Tl, as well as Sb, As, Cd, Cr, Pb, Mn, Hg, Ni, Se, Ag, and V (US Environmental Protection Agency, 2021).

### 2.2 Field Collection and Sample Processing

#### 2.2.1 Dust Traps

Eighteen passive dust traps, designed to capture multi‐month aggregate vertical wet and dry deposition, were deployed for two sampling periods (Fall 2018 through Spring 2019, and Spring 2019 through Fall 2019) throughout the Salt Lake Valley (see Table 1 for site locations and description, Table S1 in Supporting Information S1 for deployment dates, and Figure 1 for site locations). These dust traps are ideal for evaluating dust deposited at a site and estimating total dust exposure for individuals, but cannot be used to differentiate between dust deposited during dust events and dust deposited from background deposition.

**Table 1:** Site Location, Elevation, Sampler Height, and Grouping

Site ID	Site name	Sampling media	Site intent	Latitude	Longitude	Height (m AGL)	Elevation (m ASL)	Group
HW	UDAQ Hawthorne	Dust	Community exposure	40.734460	−111.872210	3.0	1,297	East
SHP	Sugarhouse Park	Dust	Community exposure	40.721045	−111.851163	1.5	1,353	East
FDTD	SLC Fire Department Training Division	Dust	High elevation	40.735913	−111.945297	27.5	1,305	High
GFRC	Gene Fullmer Recreation Center	Dust	High elevation	40.605743	−111.946709	10.1	1,347	High
TSC	UDAQ Tech Support Center	Dust	High elevation	40.776840	−111.946060		1,270	High
WS	City and County Building, Washington Sq.	Dust	High elevation	40.759377	−111.886800	33.0	1,330	High
A1	UDAQ Antelope Island	Dust	Playa signal	41.039300	−112.231300	2.5	1,351	Lake
HyS	Saline	Dust	Playa signal	41.254569	−112.496458	1.5	1,266	Lake
BV	UDAQ Bountiful Viewmont	Dust	Community exposure	40.903000	−111.884500	3.0	1,333	North
HyG	Goggin Drain	Dust	Urban‐playa interface	40.816667	−112.100000	1.5	1,284	North
O2	UDAQ Ogden #2	Dust	Community exposure	41.207000	−111.975100	4.1	1,316	North
SA	UDAQ Saltair	Dust	Urban‐playa interface	40.806100	−112.049700	2.5	1,284	North
NEF	Northeast Farmington causeway	Dust	Playa signal	41.089595	−112.162879	1.5	1,278	Playa
HyL	Lakeside	Dust	Playa signal	41.223008	−112.853097	1.5	1,272	Playa
HySWF	Southwest Farmington causeway	Dust	Playa signal	41.066159	−112.230505	1.5	1,288	Playa
H3	UDAQ Herriman	Dust	Community exposure	40.496400	−112.036310	3.0	1,541	South
LEHI	Residential Lehi	Dust	Community exposure	40.378276	−111.891064	1.5	1,357	South
AMC	UDAQ Air Monitoring Center	Dust	Community exposure	40.711800	−111.961200	3.0	1,292	West
ROSE	Residential Rose Park	Dust	Community exposure	40.791212	−111.945276	1.5	1,284	West
AV1	Bonneville bench AV Horizon 1	Vesicular horizon	Bonneville Bench Sediments	41.202010	−112.891040	0.0	1,483	
AV2	Bonneville bench AV Horizon 2	Vesicular horizon	Bonneville Bench Sediments	41.202470	−112.891820	0.0	1,494	
HyLD	Hynek Lakeside dune	Surface sediment		41.222977	−112.853364	0.0	1,272	
RVI	Ranch vegetated island	Surface sediment		40.929270	−112.158640	0.0	1,277	
FN.S	Farmington north	Surface sediment		41.091630	−112.165810	0.0	1,275	
FS.S	Farmington south	Surface sediment		41.086920	−112.160350	0.0	1,278	
FWMA.S	Farmington Waterfowl Management Area	Surface sediment		40.956970	−111.932260	0.0	1,276	
HyLP.S	Hynek Lakeside playa	Surface sediment		41.225880	−112.854910	0.0	1,275	
HySB.S	Hynek Saline beach	Surface sediment		41.301990	−112.516910	0.0	1,272	
HySP.S	Hynek Saline playa	Surface sediment		41.249840	−112.497610	0.0	1,275	
NP.S	North playa near UT Test and Training Range	Surface sediment		41.202470	−112.951520	0.0	1,276	
RDF.S	Ranch depression flakes	Surface sediment		40.921530	−112.192780	0.0	1,275	
RP1.S	Ranch playa 1	Surface sediment		40.920680	−112.132930	0.0	1,277	
RP2.S	Ranch playa 2	Surface sediment		40.926520	−112.150570	0.0	1,277	

Dust trap locations capture a gradient in human land use intensity, where the northwest sites were more isolated from anthropogenic and industrial activity, and southeast sites were more vulnerable to a variety anthropogenic and industrial inputs, representing neighborhood exposures (Figure 1). Sites within the populated parts of the Salt Lake Valley were selected to be at common exposure points like public parks (e.g., SHP), schools (e.g., HW, H3, and BV) and neighborhoods (e.g., ROSE). Some sites were selected to be higher elevation (situated on ridges or building tops, e.g., FDTD, WS, GFRC, and TSC) to isolate regional contributions from localized contributions. Many sites were co‐located with Utah Division of Air Quality Monitoring sites (e.g., O2, TSC, HW, and H3).

Among our lake proximal sites, three were situated on the playa (HyL, NEF, and HySWF) and two were situated on lake‐proximal paleo Lake Bonneville benches above the modern lakeshore (Antelope (A1) and Saline (HyS)). The geochemical differences (particularly strontium isotopes) between these two classes of sites helps illuminate the modern playa contributions to dust relative to potential contributions of paleo Lake Bonneville sediments to regional dust. Saline is located close to gravel pits used for mining the paleo‐shoreline, and the exposed playa area adjacent to the sampler is much smaller compared to our Lakeside or Farmington Bay sites. The Antelope site is not affected by gravel mining and is proximal to a greater expanse of dry playa.

Following Reheis et al. (1995), dust was collected in Teflon‐coated Bundt cake pans filled to 2.5 cm below the lip of cake pan with a mix of pre‐cleaned colored and clear glass marbles. Bird deterrent stainless steel spikes were installed on the surface of the cake pans. The dust traps were either mounted to a polyvinyl chloride pipe attached to a steel t‐post or to the rooftops of urban buildings (Table 1).

For analysis, the contents of the Bundt pans including the glass marbles were poured and rinsed into pre‐cleaned polypropylene pans using ultra‐pure deionized water. Large materials such as bugs, leaves, or feathers were removed. The marbles were rinsed using ∼1 L of ultra‐pure deionized water to remove any remaining material collected during deployment. The liquid contents of the pan were poured into a 3 L polypropylene bottle, and filled to a total volume of 3 L using ultra‐pure DI water. The bottles were shaken to homogenize the solution and undissolved solid material in the sample was allowed 3 days to settle to the bottom of the bottle. Two liters were decanted from the bottle to entrain as little particulate matter as possible. The final one L split was wet sieved through non‐metallic 63 μm sieves into glass beakers. The slurry was dried in a 60°C oven. Once dry, the samples were flaked off of the beakers into glass vials for analysis. We note that the rinsing, settling and decanting process may contribute to dissolution of soluble and/or bioavailable cations and metals. This means that our results may represent the lower bound on the relative abundance of bioavailable Na, Ca, Li, and As, among other soluble elements.

The <63 μm threshold is common operational definition for sediment source tracking studies because it matches the sand to silt size cutoff. Restricting the sediments to smaller grain sizes ensures sample homogeneity and mitigates the outsized impact of coarser mineral grains on chemistry (i.e., quartz dilution). It also focuses analyses on the size fraction where bioavailable metals are often sorbed.

#### 2.2.2 Sediments

Surface sediments were collected using a 30‐grab spoke method (Gold et al., 2019; Hess et al., 2019; Smith et al., 2006), where 5 grabs were collected along each of 6 spokes, with 5 paces between each grab. Only the top 0–2 cm were sampled using a clean plastic trowel. Material was collected into a clean plastic tray.

The bulk sediment samples were air dried for 1 week. Once dry, the samples were homogenized and 350 g analysis splits were produced. Non‐metallic nested sieves were used to separate the sediment into >250, 250–125, 125–63, and <63 μm fractions. The samples were wet sieved into glass beakers using ultra‐pure DI water, where they were air dried then transferred to sample cups for analysis.

Among our sediment samples we collected and analyzed fine‐grained vesicular soil horizons (AV1 and AV2). These soil horizons were sampled uphill from the Lakeside (HyL) sampler by about 200 m, on a paleo shoreline of Lake Bonneville. Vesicular horizons, which are fine grained, near surface layers characteristic of arid environments have been documented as composed of deposited dust (Reheis et al., 2009), though the dust may be hundreds to thousands of years old. We interpret this sample as representative of a natural dust trap of unknown age, and provides a contrast to the contents of our bundt dust traps.

### 2.3 Sample Analysis

#### 2.3.1 Geochemical Analysis

We subjected the <63 μm size fraction of the dust trap collections and the surface sediment collections to a cold leach (22°C) for 24 hr in 0.8 M HNO_3_. This acid extraction, while stronger than other weaker alternatives used to evaluate bioavailable proportions of metals (e.g., ammonium acetate buffers), represents the upper end estimate of possible exposure loads of metals and many elements. Geochemical analysis of the supernatant for elemental concentrations was performed in the ICP‐MS Metals and Strontium Isotope Facility of the Geology and Geophysics Department at the University of Utah by quadrupole ICP‐MS (Agilent 8900, Santa Clara, California, USA) using an external calibration method with internal standard.

Analysis for ^87^Sr/^86^Sr ratios was performed from the same leachate as the geochemical analysis. Strontium isotope ratios were chosen for analysis because they can be used to fingerprint contributions of GSL playa sediments to dust. This is possible because of the large isotopic separation between the strontium isotope ratios of GSL playa sediments and sediments and dust derived from other regional playas and sources (e.g., Carling et al., 2020). Instrumental analysis was performed in the University of Utah Trace Metals Lab by multi‐collector ICP‐MS (Neptune Plus, Thermo Finnigan, Bremen, Germany) after chromatographic purification of Sr (PrepFast, Elemental Scientific, Omaha, Nebraska, USA). Data are available in Blakowski et al. (2022).

#### 2.3.2 Data Quality Assurance

To prepare the data set for analysis, we subjected the data to a multi‐step quality assurance process, which included examining blanks and evaluating reproducibility of duplicates. The cleaning method and instrument blanks indicated low or non‐detect levels of all elements. Most elements measured in the 26 duplicated samples agreed well, suggesting high confidence in our analytical method (Figure S1 in Supporting Information S1). Elements of concern in terms of analytical precision included Selenium (Se) and Antimony (Sb), which were not included in further analyses.

The relative abundances Ce and Er in our dust traps, and particularly their ratios with other REEs (e.g., La/Ce) and values when normalized to chondrite, suggest Ce and Er contamination in our samples. We did not observe these issues in our sediment samples, suggesting that contamination may have come from either the marbles or bundt pan. Any weathering processes causing contamination could not have been captured by our method blanks. These elements were removed from further analysis.

#### 2.3.3 Dust Flux and Mass Weighted Average Calculations

We calculated the dust flux (*F*_*x*_) for each element (*x*) as the product of the mass of the sample that is <63 μm (*M*_*s*_, <63), the relative abundance of the element in the sample (*RA*_*x*_), divided by the sampler area (*SA*) and the sampling interval (*I*) (Equation 1). This yielded element‐specific fluxes in units of mg m^−2^ day^−1^. Values from literature data (Goodman et al., 2019; Heindel et al., 2020) were converted to the same units as our data for contextualization.

(1) $$F_{x}=\frac{M_{s,<63}*RA_{x}}{SA*I}$$

We chose to calculate the mass‐weighted average metal relative abundances for our analyses for the portion of sample with grain sizes <63 μm (Equation 2). This is because we sampled only two intervals, and the first sampling period was of variable length, so we could not discern which patterns arose due to seasonal variability and which arose due to deployment‐specific conditions. Mass weighted average relative abundances $\left(\bar{RA_{x}}\right)$ were calculated as the sum of the products of sample masses (*M*_*i*,<63_) and element relative abundances (*RA*_*x*,*i*_) across the two sampling intervals (*i*), divided by the sum of the sample masses (*M*_*i*,<63_).

(2) $$\bar{RA_{x}}=\frac{\sum RA_{x,i}*M_{i,<63}}{\sum M_{i,<63}}$$

### 2.4 EPA Regional Screening Levels

We cannot fully constrain the health risks of dust exposure because we did not measure actual exposure to trace metals or consider likely health outcomes of ingesting or inhaling dust in this study. Likewise, our samplers, which measure dust deposition, likely underrepresent airborne dust exposure relative to samplers that target horizontal flux or atmospheric concentration. However, to start to understand the health risks of trace metal pollution exposure via airborne particulate matter, we compared our data to Regional Screening Levels for soil (RSLs; Table 2) set by the EPA. Some of these trace elements are considered “Priority Pollutants” by the EPA. Priority pollutants are those that are regulated by EPA, and for which they have developed analytical test methods. For more details and considerations in evaluating dust relative to soil RSLs, see Text S1 in Supporting Information S1.

**Table 2:** Summarizing Environmental Protection Agency (EPA) Regional Screening Levels (RSLs) for Soil and Major Anthropogenic Sources of Metals and Metalloids, 13 of Which Are Designated by the EPA as Priority Pollutants (Denoted With Asterisks)

Element	Residential RSL (mg/kg)	Industrial RSL (mg/kg)	Anthropogenic sources	Common forms in wastes	References
Li	16	234	Fossil fuel combustion and battery manufacture		Moreno et al. (2008)
Be*	16	229	Nuclear industry, electronics industry, and fossil fuel combustion	Be alloys, Be metal, and Be(OH)_2_	Fishbein (1981) and Taylor et al. (2003)
B	1,560	23,300	Fossil fuel combustion and sewage sludge		Akar (2007)
Al	7,740	112,000	Fossil fuel combustion, vehicle emissions, soil dust		Moreno et al. (2013)
V	39	583	Fossil fuel combustion, sewage sludge, fertilizers, and soil dust		Moreno et al. (2008)
Cr*^a^	11,700; 23	175,000; 350	Metallurgy, vehicle emissions, fossil fuel combustion, landfills, plastic industry, cement industry, paint industry, wood treatment, and soil dust	Cr metal, Cr oxides (oxyanions), and Cr^3+^ complexes with organic/inorganic ligands	Fishbein (1981), Moreno et al. (2013), and Saha et al. (2011)
Mn	183	2,560	Vehicle emissions, metallurgy, and soil dust		Moreno et al. (2013) and Sweet et al. (1993)
Fe	5,480	81,800	Vehicle emissions, fossil fuel combustion, and soil dust		Matsui et al. (2018) and Moreno et al. (2013)
Co	2.3	35	Fossil fuel combustion, mining and smelting, metallurgy, vehicle emissions, fertilizers, waste incineration, and electronic waste recycling		Xue et al. (2021)
Ni*	83	1,160	Mining and smelting, metallurgy, electronics industry, wood treatment, landfills, swine manure, pesticides, paint industry, and vehicle emissions	Ni metal, Ni^2+^ ions, Ni amines, and alloys	Fishbein (1981) and Chow et al. (2004)
Cu*	313	4,670	Mining and smelting, metallurgy, electronics industry, wood treatment, landfills, swine manure, pesticides, paint industry, and vehicle emissions	Cu metal, Cu oxides, Cu humic complexes, alloys, and Cu^2+^ ions	Y. Huang et al. (2018) and Moreno et al. (2013)
Zn*	2,350	35,000	Mining and smelting, metallurgy, textiles manufacture, electronics industry, pesticides, vehicle emissions, tire wear, lubricants, waste incineration, landfills, and sewage sludge	Zn metal, Zn^2+^ ions, Zn oxides and carbonates, and alloys	Salim Akhter and Madany (1993), Fergusson and Kim (1991), X. Huang et al. (1994), Moffet et al. (2008), Moreno et al. (2013), Morishita et al. (2006), and Wuana and Okieimen (2011)
As*	0.68	3	Fossil fuel combustion, mining and smelting, herbicides, pesticides, poultry manure, metallurgy, and wood treatment	As oxides (oxyanions), organo‐metallic forms, methylarsinic acid, and dimethylarsinic acid	Fishbein (1981), Moreno et al. (2013), and Xie et al. (2006)
Se*	39	584	Mining and smelting, fossil fuel combustion, and irrigation waters	Se oxides (oxyanions), and Se‐organic complexes	Chow et al. (2004), Moreno et al. (2013), Wen and Carignan (2007), and Xie et al. (2006)
Sr	4,690	70,100	Fertilizers, fossil fuel combustion, nuclear industry, and soil dust		Hosono et al. (2007)
Zr	0.63	9.3	Vehicle emissions and soil dust		Moreno et al. (2013)
Mo	39	584	Mining and smelting, fossil fuel combustion, waste incineration, fertilizers, and soil dust		Barron et al. (2009), Frascoli and Hudson‐Edwards (2018), and Wong et al. (2021)
Ag*	39	584	Mining and smelting, fossil fuel combustion	Ag metal, Ag‐CN complexes, Ag halides, and Ag thiosulfates	Lee et al. (1994)
Cd*	7.1	98	Mining and smelting, metallurgy, plastic industry, electronics industry, battery manufacture, landfills, sewage sludge, fertilizers, waste incineration, paint industry, tire wear, and lubricants	Cd^2+^ ions, Cd halides and oxides, Cd‐CN complexes, and Cd(OH)_2_ sludge	Salim Akhter and Madany (1993), Fergusson and Kim (1991), Fishbein (1981), Y. Huang et al. (2018), Morishita et al. (2006), Rahman et al. (2019), and Wuana and Okieimen (2011)
Sb*	3.1	47	Electronics industry, metallurgy, mining and smelting, vehicle emissions, brake wear, and waste incineration	Sb^3+^ ions and Sb oxides and halides	Chow et al. (2004), Dietl et al. (1997), X. Huang et al. (1994), and Moreno et al. (2013)
Ba	1,530	21,700	Mining and smelting, fossil fuel combustion, and soil dust		Pragst et al. (2017)
Hg*^b^	1.1	4.6	Fossil fuel combustion, mining and smelting, electrolysis industry, plastic industry, landfills, paper/pulp industry, and fungicides	Organo‐Hg complexes, Hg halides and oxides, and Hg^2+^, Hg^0^	Sherman et al. (2015) and Wuana and Okieimen (2011)
Tl*	0.08	1.2	Mining and smelting, metallurgy, fossil fuel combustion, electronics industry, cement industry, and waste incineration	Tl halides and Tl‐CN complexes	Karbowska (2016), Migaszewski and Gałuszka (2021), and Vanĕk et al. (2016)
Pb*	400	800	Mining and smelting, metallurgy, fossil fuel combustion, plumbing, battery manufacture, sewage sludge, landfills, waste incineration, pesticides, and paint industry	Pb metal, Pb oxides and carbonates, and Pb metal‐oxyanion complexes	Moffet et al. (2008), Morishita et al. (2006), and Wuana and Okieimen (2011)
La	0.39	5.8	Fossil fuel combustion		Kulkarni et al. (2007) and Moreno et al. (2008)
U	1.6	23	Soil dust, mining and smelting, nuclear industry, and fertilizers		Takeda et al. (2006)

*Note.* Common forms and anthropogenic sources of the priority pollutants were modified from Adriano (2001, Table 1.3) and from references cited in the table. Anthropogenic sources of non‐priority pollutants were summarized from Agency for Toxic Substance and Disease Registry toxicological profiles and from the cited references. *EPA as Priority Pollutants.

^a^ We report RSLs for both Cr(III) and Cr(VI) because humans can be exposed to either form (although Cr(VI) is likely more toxic) (Kimbrough et al., 1999).

^b^ The most common way humans are exposed to Hg is by eating seafood that contains methylmercury (MeHg). However, human exposure to Hg in industrial settings is attributed to inhalation of elemental Hg vapors (Clarkson, 2002). Hence, we report RSLs and anthropogenic sources of elemental Hg.

### 2.5 Data Analysis

#### 2.5.1 Principal Component Analysis

We used a principal component analysis (PCA) of dust trace element relative abundances to identify groupings in our samples. This method of variance explanation is helpful for determining the sources of industrial particulates and metals bound to fugitive dust (e.g., Goodman et al., 2019; Heindel et al., 2020). The PCA included our surface sediment and vesicular horizon data with grain sizes <63 μm (*n* = 4) as well as all bundt pan collected dust (*n* = 38) from this study. We only included surface sediments in the finest size fraction because relative abundance of trace metals can depend on grain size due to differences in surface area to volume ratios. Because PCA requires a complete data set (no non‐detect or screened values permitted) we removed all elements that had even one sample with a reported value below the detection limit. Elements we removed from the analysis included Mo, Ag, and Sc. This left more than 30 trace elements available for inclusion in the analysis. The trace element data were normalized by element so as to equalize the means and variances among elements. We specified that the PCA should be composed of two PCs, as additional PCs did not explain substantially more variance in the data set.

#### 2.5.2 Linear Regression

We performed simple and multiple linear regressions to test specific hypotheses about trace element sources as well as relationships between community demographics and trace metal abundance in our data set. When comparing among regression models, the akaike information criterion (AIC), R^2^, and p‐values were used to assess model performance.

We tested specific hypotheses with regression analyses. We tested whether Tl could be traced to Cu mining, smelting and concentrating using regressions with Cu and Mo, following the major elements present in the Bingham Copper Mine. We also tested whether the majority of As in urban dust traps comes from GSL dust, as posited by recent articles detailing As‐specific public health concerns associated with the desiccation of the GSL (Flavelle, 2022; Kafanov, 2021; Spencer, 2021). Strontium isotope ratios, as well as linear combinations of elements concentrated on the playa, including Mg, Li, Ca, and Sr can collectively trace playa dust contributions (Table S2 in Supporting Information S1). If all As in our urban dust traps comes from GSL playas, then ^87^Sr/^86^Sr, or some linear combination of Li, Mg, Ca, and/or Sr, should indicate a significant positive correlation with As.

We tested relationships of suites of elements to demographic information. For the linear regressions performed as part of the demographics analysis, all data used came from the 2019 census (United States Census Bureau, 2020) and regressions were performed at the zip code scale. The sampler was assigned to the zip code it was located in, and linked to the demographic information associated with the zip code.

## 3 Results

### 3.1 Dust Mass Flux and Particle Size

Our samplers collected between 10 and 140 mg m^−2^ day^−1^ of fine grained dust, where fine grained dust included any material with grain sizes <63 μm, depending on the site and season (Figure S2 in Supporting Information S1). Sites situated around Farmington Bay (e.g., NEF and HySWF) and between the GSL and downtown (e.g., SA and BV) collected the most dust (85–140 mg m^−2^ day^−1^). City sites to the south near the western boundary of developed land (e.g., H3, LEHI) collected more dust than sites situated within the city. Sites situated within city centers exhibited the lowest dust fluxes.

Dust particle size distribution indicated a higher proportion of coarse grained material at playa and playa‐adjacent sampling sites, and a higher proportion of fine grained material in the city, especially at higher elevation rooftop samplers within the city (Figure S3 in Supporting Information S1). In this study we used the percent of total dust mass <63 μm as our proxy for particle size distribution. On average, about 63% of the mass of dust in our collectors was from dust smaller than 63 μm. Between 20% and 40% of dust mass deposited to our ground‐level collectors near Farmington Bay, which is often dry, was <63 μm. The mass of dust <63 μm at ground level sites in the Salt Lake Valley was between 35% and 75%. Finally, upwards of 80% of the dust mass collected at the highest elevation collectors in the city (WS, GFRC, and FDTD) was dust <63 μm.

### 3.2 ^87^Sr/^86^Sr

**Figure 2:**
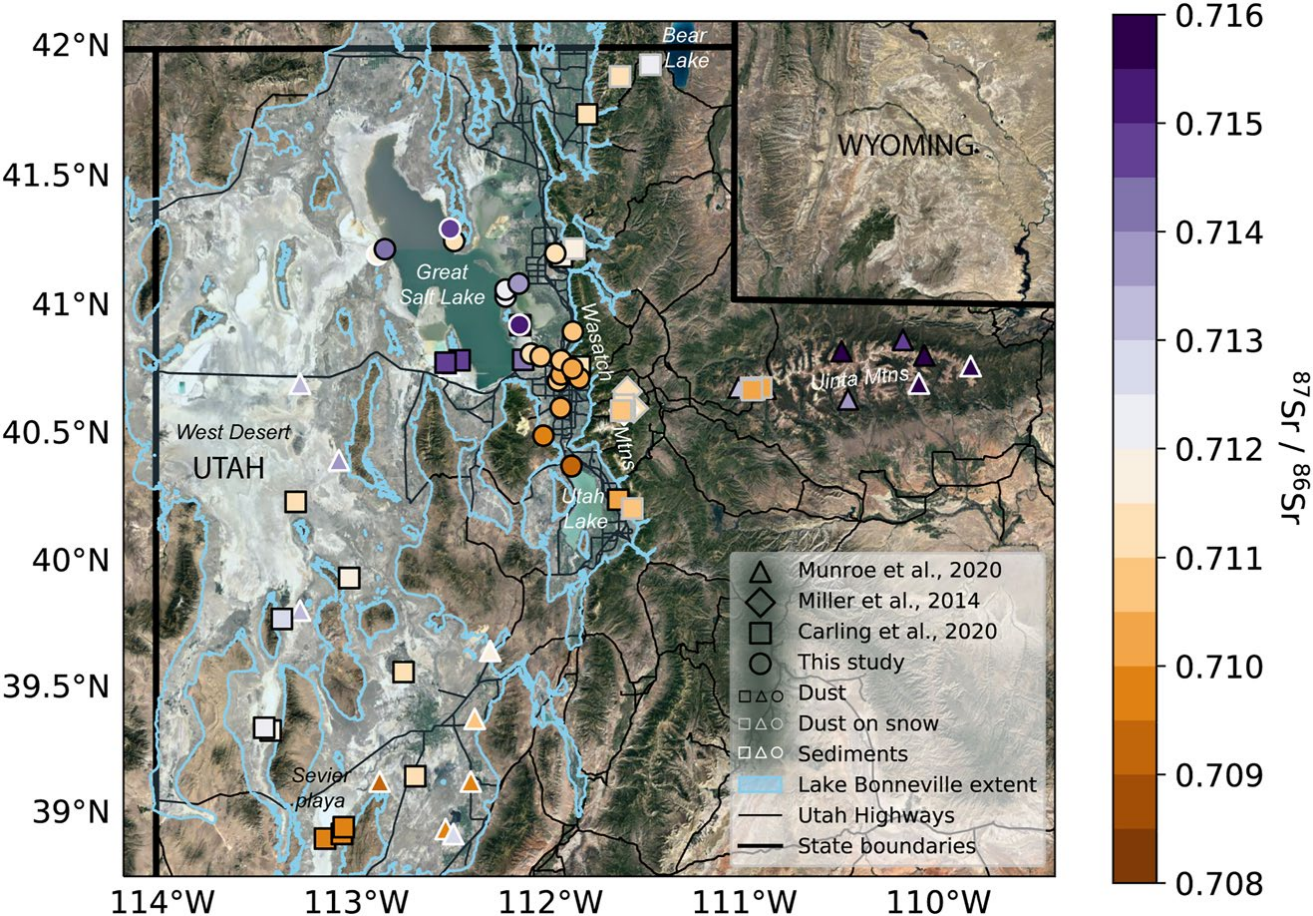
The spatial distribution of strontium isotope ratios (^87^Sr/^86^Sr). Higher strontium isotope ratios are characteristic of the Great Salt Lake sediments, while lower strontium isotope ratios are characteristic of the Sevier Dry Lake playas. Markers indicating source sediment collections have a gray outline, while markers indicating dust collections have a black outline.

We measured the highest ^87^Sr/^86^Sr (∼0.715) in dust deposited at sites proximal to the GSL and its playa (Figure 2), and especially at Lakeside (HyL) and North East Farmington (NEF). These matched isotope ratios observed in playa sediments. Isotope ratios in the dust samples collected for this study decreased with distance from GSL, with northerly city sites exhibiting strontium isotope ratios of greater than 0.711 and southerly sites indicating strontium isotope ratios of 0.710 or less. Rooftop sites indicated similar ^87^Sr/^86^Sr ratios as proximal lower elevation dust collectors. Two lake‐proximal samples differed from our observed north‐south pattern. Saline (HyS), located to the east of the north arm of GSL, and Antelope (A1) located on the northern end of Antelope Island indicated lower ^87^Sr/^86^Sr than other samples on GSL playa. These samples indicated strontium isotope ratios similar to urban or exurban samples situated close to GSL, like Ogden (O2), Saltair (SA), Bountiful (BV), and Rose Park (ROSE).

The surface sediment samples from around GSL tended to indicate high ^87^Sr/^86^Sr, similar to results from “playa” samplers like Lakeside (HyL) and NEF. However, samples collected from two soil vesicular horizons (AV1 and AV2) on a hillside near the Lakeside site indicated lower strontium isotope ratios, similar to dust samples from Antelope (A1), South West Farmington (HySWF), and Saline (HyS). While the observed strontium isotope ratios are higher than many urban samples, they are substantially lower than proximal lake bed surface sediment samples (HyLD and HyLP.S) and Lakeside (HyL) dust samples.

### 3.3 Trace Element Relative Abundances

Our playa dust samples (from HyL, NEF, and HySWF) were enriched relative to upper continental crust in elements associated with evaporite and carbonate minerals, including Li, B, Na, and Sr (Figure S4 in Supporting Information S1; Rudnick & Gao, 2003). Playa dust also indicated enrichments of anthropogenically associated trace elements like Ni, Pb, As, Zn, Cu, and Cd. Playa‐proximal Bonneville bench dust sites indicated fewer enrichments in elements associated with evaporite and carbonate minerals (Li, Na, and B), and greater enrichments in anthropogenically associated trace elements including Pb, As, Zn, Cu, and Cd. For urban dust sites, most did not indicate enrichments in elements associated with evaporite and carbonate minerals. Instead, we observed enrichments of anthropogenically associated trace metals like Ni, Pb, As, Zn, Cu, and Cd. The enrichments were highest in most of these elements (except Ni) at the highest elevation samplers (WS, GFRC, FDTD, and TSC).

**Figure 3:**
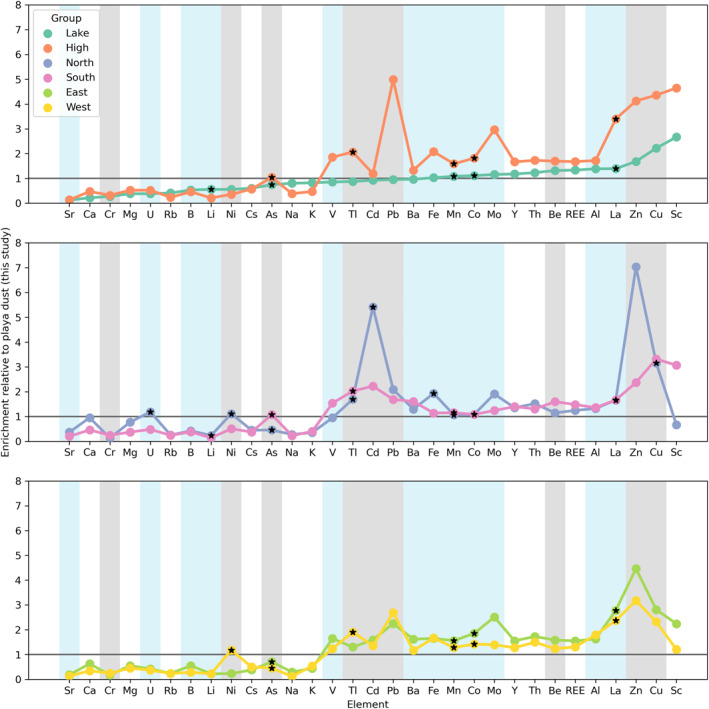
Mass‐weighted enrichments of dust in different regional groups relative to playa dust collections. Vertical blue shading indicates the element has a Regional Screening Level (RSL), and vertical gray shading indicates the element is a Priority Pollutant. Elements are ordered by the enrichment of “lake” category samplers. If any sample composing the group average indicated an exceedance of an Environmental Protection Agency RSL, that mean value was marked with a black star. The organization of groupings into the panels is to highlight similarities and differences between (a) lake proximal and high elevation sites, (b) northerly and southerly sites, and (c) sites on the east and west side of downtown.

When the lake‐proximal Bonneville bench dust sample relative abundances were compared with the mass weighted multisample average relative abundances in playa dust (HyL, NEF, and HySWF), we observed enrichments in Fe, Mn, Co, Mo, Y, Th, REEs, Be, La, Al, Zn, and Cu (Figure 3). Urban dust collections indicated the same enrichments, along with As, Tl, V, Cd, Ba, and Pb at the highest elevation samplers, as well as Ni in Bountiful and West Salt Lake City (BV, AMC, and ROSE), and U in Bountiful (BV). Based on our data (Figure 3a) it is unlikely that unaltered dust from the GSL playa is deposited in Salt Lake City, because the metals enrichments are so different. However, with a couple of exceptions (Cd and Zn), we see little evidence of a longitudinal gradient in the overall relative abundances of metals in our data set (Figure 3b). Finally, we observe similar enrichments for the east and west side of Salt Lake City, with the east side tending to exhibit higher relative abundances for all metals besides Ni, Tl, and Pb (Figure 3c).

**Figure 4:**
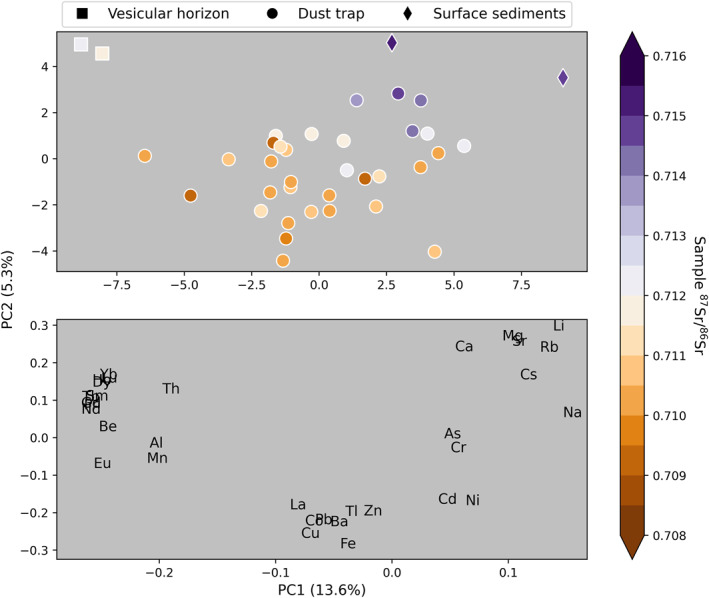
A principal component analysis of dust traps and source sediments from this data set, with dots colored by strontium isotope ratio, to reveal how sample grouping based on strontium isotope ratios compares with grouping based on geochemical makeup. We find that both strontium isotope ratios and geochemistry sort samples in a similar way, with samples from near the Great Salt Lake geochemically and isotopically distinct from those within the city. The first two PCs explain a combined 20% of the variance in the geochemistry data. Note that a subset of surface sediment data was included in the analysis because not all surface sediment samples had substantial fractions <63 μm available for geochemical analysis.

A PCA provides a second line of inquiry for evaluating the relationship between the GSL playa and urban dust. In our PCA the first two principal components, which together explain a total of about 19% of the variance in the data set, sort the data into three main groupings (Figure 4). Playa and lake shore, and to a lesser degree, northerly community exposure sites (O2 and BV), indicate higher normalized relative abundances of elements associated with carbonate and evaporite minerals (Sr, Li, Ca, Mg, Rb, Na, and Cs). This group had high PC1 and high PC2 scores and exhibited higher strontium isotope ratios. Dust from high elevation sites and the vesicular horizons contain higher normalized relative abundances of REEs, Al, Mg, Th, and Be, scoring low on PC1. Finally, most community‐exposure level urban dust contains higher normalized relative abundances of La, Zn, Tl, Ba, Fe, Pb, Co, and Cu, indicated by low PC2 scores.

The priority pollutants that we measured that exceed EPA residential RSLs at one or more community exposure sites are As, Cd, Ni, and Tl. Relative abundances of As and Tl in dust exceeded the EPA RSL at all and most sites, respectively (Figure 3 and Figure S5a in Supporting Information S1). Cobalt (Co), La, Mn, and U also exceeded the residential RSL at in dust one or more community exposure sites. Relative abundances of La, Mn, and Co exceeded the EPA residential RSL at all or most dust sampling sites (Figure 3 and Figure S5b in Supporting Information S1). Dust traps situated at higher elevation, such as on roofs of buildings, had a greater proportion of trace elements that exceeded residential RSLs (median 23%) than dust traps placed on the lakeshore (16.7%) and those in the city but positioned closer to the ground (16.7%).

#### 3.3.1 Specific Trace Element Results

Among the trace elements measured, La, Tl, Co, Mo, and As are of particular interest in this region. The specific analyses performed to evaluate their spatial patterns and illuminate their potential sources is presented in this section.

**Figure 5:**
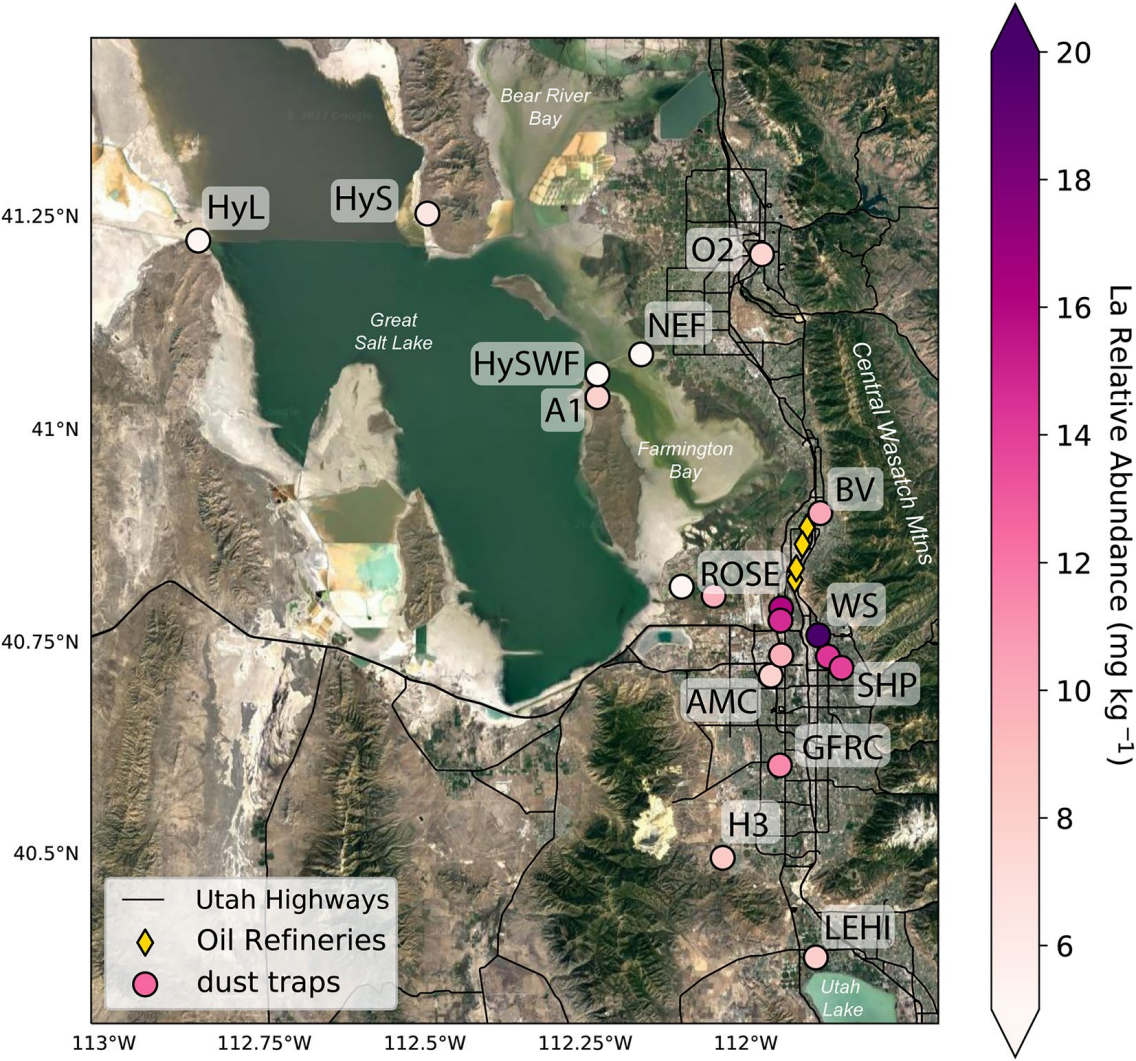
Spatial distribution of flux‐weighted La relative abundances with the locations of Salt Lake Valley oil refineries indicated by yellow diamonds.

Lanthanum concentrations exceeded the residential RSL for soil in every dust sample collected in this study (Figure 5). The highest relative abundances of La occurred in ROSE (18 mg kg^−1^) and on the city and county building (WS, 42 mg kg^−1^). Sites near downtown indicated elevated levels of La (∼14 mg kg^−1^), with relative abundances decreasing with distance from downtown. Higher elevation sites (GFRC and FDTD) indicated higher relative abundances relative to their distance from downtown.

Thallium exceeded the EPA residential RSL at nine of our dust collection sites. We observed higher relative abundances at higher elevation sites (WS, FDTD, and GFRC), in exurban communities (H3, LEHI, and SA), and lower‐income, less white communities (AMC and ROSE). In our data set, a multiple linear regression of flux‐weighted mean Cu and Mo best explain the variance in flux‐weighted mean Tl (*R* ^2^ = 0.60, *p* < 0.001, AIC = −81.05). Flux weighted mean Cu (*R* ^2^ = 0.51, *p* < 0.001, AIC = −79.47, shown in Figure S6 in Supporting Information S1), Mo (*R* ^2^ = 0.385, *p* = 0.0046) and Sb (*R* ^2^ = 0.19, *p* = 0.061) also can significantly predict Tl in our dust samples.

**Figure 6:**
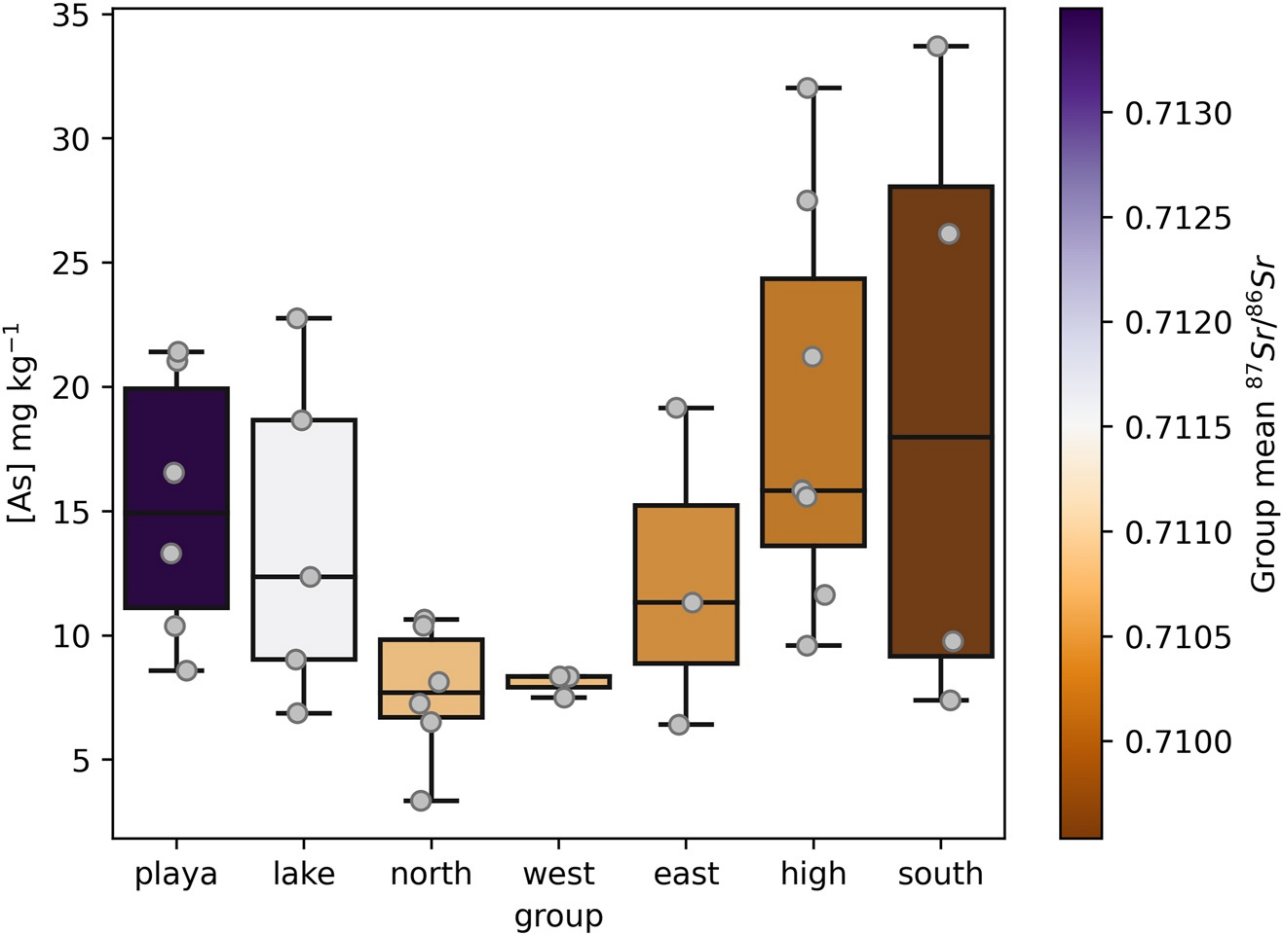
Arsenic distributions in our samples grouped by sampler region and colored by the mean strontium isotope ratio for the group. Individual samples are plotted on top of the boxes.

Arsenic concentrations exceeded the residential RSL for soil in every dust sample collected in this study by a median of 18 times, and as much as 35 times. Arsenic relative abundances and fluxes were highest at high income exurban sites (LEHI and H3), and lowest in low‐income urban sites. Dust traps on buildings and in the southern part of our study area included more As than found in our lake‐proximal samplers. However, the As is not correlated with strontium isotope ratios (Figure 6), nor any other combination of “playa” element (e.g., Li, Ca, Na, and Sr). Instead the only trace elements that produce moderate correlations with As among the dust traps are Ba and V (positive). The mass‐weighted mean As and V relative abundances exhibited a correlation coefficient of 64.5%.

### 3.4 Interaction Between Trace Metals Abundances and Ethnicity and Income Demographics

Across all community exposure sites (SHP, ROSE, HW, BV, O2, H3, AMC, and LEHI), we found the highest fluxes of trace metals at wealthy exurban sites (H3, LEHI, and BV), due to their high dust fluxes. Higher income sites, both urban and exurban, had higher concentrations of As and V than lower income sites, with zip code median income explaining 68% of the variance in the mass‐weighted mean relative abundance of As (*p* = 0.0225).

Other trace elements (Pb, Tl, and Ni) indicated higher relative abundances and were more likely to exceed EPA RSLs in lower income communities with higher proportions of Hispanic residents relative to all other sites, including the two other exposure sites on the east side of Salt Lake City (HW and SHP; Figure 3). Although Pb did not exceed the EPA residential RSL at any site, there is no safe level of Pb. Of these elements, Pb indicated the strongest relationship between neighborhood race or ethnicity and relative abundance across all eight sampling sites, where neighborhoods with lower proportions of white residents and higher proportions of Hispanic residents tended to have dust with higher relative abundances of Pb, though the relationship was not significant.

Among the four exposure sites within Salt Lake City which are the most comparable sites for understanding the effect of neighborhood and race or ethnicity on metals exposure from particulate matter, we found a significant relationship between concentrations of Tl in dust and the proportion of people in the zipcode with Hispanic ethnicity (*p* = 0.0456, *R* ^2^ = 0.67). Neither Ni nor Pb indicated a significant relationship at this refined spatial scale.

## 4 Discussion

### 4.1 Urban Dust Contains Fugitive Dust From Local Soils and Anthropogenic Particulate Pollution

We interpret depositional dust fluxes and grain size patterns, Sr isotope ratios, and PCA results, to inform the local and regional sources of dust in our Salt Lake Valley dust collectors. The grain size proportion analysis informs the prevalence of local dust transport (e.g., road or construction dust, see Heindel et al. (2020)) to total dust deposition at community exposure sites in the city, while the strontium isotope data and the PCA results provide multi‐constituent estimates of dust and particulate pollution provenance. Because of the known heterogeneous contamination of GSL sediments (Perry et al., 2019), evaluating the potential contributions of GSL playa to urban dust informs the recent widespread concern that these source sediments may contribute elevated levels of toxic or Priority Pollutant metals (Kafanov, 2021; Spencer, 2021).

#### 4.1.1 Fugitive Dust Contributes to Dust Loads in Urban Areas

The depositional dust fluxes we observed (10–140 mg m^−2^ day^−1^) were similar to fluxes reported by Goodman et al. (2019) for the same region (65–95 mg m^−2^ day^−1^), and higher than dust fluxes at Colorado front range plains sites reported in Heindel et al. (2020) (∼52 mg m^−2^ day^−1^) using similar methods. We found higher dust fluxes and a lower proportion of dust <63 μm at sites close to the GSL and outside of the urban area (e.g., SA, BV, H3, and LEHI) and lower dust fluxes and higher proportions of dust <63 μm in high‐elevation rooftop samplers within the urban core (e.g., FDTD, WS, and GRFC). These spatial patterns are consistent with established physical relationships between grain size, wind speed, and transport distance, where larger grains tend to be transported shorter distances, lower in the atmosphere (Lawrence & Neff, 2009). Because dust grains transported from local playas to the Wasatch Mountains during strong windstorms have been observed to be large (∼20–30 μm; Nicoll et al., 2020), we expect that under most conditions these grains may not make it far from the playa. This is compounded by the wind deceleration caused by the increased land surface roughness in cities relative to playa and rangeland (Meier et al., 2022). When high wind events transport dust from outside of the city to the city edge, the abrupt change of surface roughness causes rapid wind deceleration resulting in detrainment of large particulates (Zhang et al., 2001) at the edge of the urban area. Fine grained material remains entrained, and can be deposited within the city, in the Wasatch Mountains (Carling et al., 2020; Skiles et al., 2018) or may even be transported to the Uinta Mountains (Reynolds et al., 2010). In the northern part of the valley, this idea of rapid dust deposition on the outskirts of and near the edges of the urban zone are supported by the gradient in strontium isotope ratios observed in the northern part of the Salt Lake Valley. The highest Salt Lake Valley strontium isotope ratios are observed on the northern end of the city, with lower isotope ratios observed at more southerly sites (Figure 2), with no systematic pattern in strontium isotope ratios with sampler height.

For ground‐level urban samplers, we observed greater total depositional dust fluxes and lower proportions of dust <63 μm relative to the rooftop samplers, suggesting the importance of local dust sources (e.g., road, gravel pit, or construction dust) to human dust exposure. There are many recorded active and legacy gravel pits (Figures S2 and S3 in Supporting Information S1) that mine the sediments of paleo‐shorelines of Lake Bonneville. Finding higher dust fluxes near human disturbance is consistent with Heindel et al. (2020), where their Skywatch site, situated near active construction, exhibited higher dust fluxes than sites further from human soil disturbance. The relationships between relative abundance and sampler height for elements largely contributed by particulate pollution also supports this conclusion. For example, La, Tl, Pb, and Cu tend to be present in higher relative abundances in rooftop dust samples than low elevation dust samples. The relative enrichment of fine grained particles may arise because of the enhanced metals adsorption capacity of fine particles, the size of anthropogenically emitted, metal‐embedded particles, and/or the presence of secondary minerals which can act as adsorbents for trace metals (Bi et al., 2013). Our observations suggest that all elevations receive similar loads of particulate pollutants, but the contributions of larger‐grained soil or construction dust in lower elevation samplers dilutes the relative abundance of metals delivered on fine‐grained particulates.

We did not observe a systematic gradient in strontium isotopes with elevation as we might expect if the GSL playa was contributing a majority of regional dust to our urban dust collection sites. This suggests that regional geogenic dust, including from all regional playas as well as other sources, which has been hypothesized to contribute the majority of the strontium delivered to rooftop samplers (Carling et al., 2020), and the fugitive dust from local soils, which should provide the majority of the strontium in the community exposure samplers, have similar strontium isotope ratios. Our data in context of regional data sets provides support for this hypothesis.

#### 4.1.2 Strontium Isotope Ratios Inform Dust Provenance in Urban Areas

Strontium isotope ratios vary across the Great Basin, and can be used to trace the origin of dust. Hart et al. (2004) measured an Sr ratio of 0.71472 for strontium dissolved in GSL, and 0.72000, and 0.70990 for strontium dissolved in the Bear and Weber Rivers. The relatively higher isotope ratio observed in strontium dissolved in GSL is also present in the dust from the GSL playa (∼0.715), as compared to dust from the Sevier Dry Lake (∼0.710) and other regional playas (∼0.711 to 0.712; Carling et al., 2020; Figure 2). Strontium isotopes of city sites closer to the GSL suggest higher contributions of GSL playa dust than city sites further to the south.

Our highest strontium isotope ratios, observed at samplers on GSL playa, matched prior measurements from Carling et al. (2020) and were similar to, but lower than the average strontium isotope ratio for GSL brine (0.7174 ± 0.0004) (Jones & Faure, 1972). The similarity between the Lakeside (HyL) and NEF sites and the GSL sediments strontium isotope ratios suggest that dust at these two sites is composed of dust from lake sediments. Though the playa exhibits substantial spatial variability in most trace elements, and in many regions the sediments contain high relative abundances of anthropogenic metals like Pb and Cu (Perry et al., 2019), we suggest that dust from our playa sites may be used as indicators of approximate composition and relative abundance of trace elements that we expect to be emitted from GSL playa sediments. We do not suggest that the playa sediments represent an “unpolluted” dust source.

The Salt Lake Valley urban sites indicated similar or lower strontium isotope ratios than reported for the West Desert playa (Carling et al., 2020), dust on snow from Wasatch mountains (Miller et al., 2014), and fully acid‐digested dust deposited in the Uinta mountains in any season (Figure 2; Munroe et al., 2019). The full acid digestion for the Munroe et al. (2019) samples likely biases them toward higher isotope ratios than the other methods. The lowest isotope ratios among all data sets occurred in southern Salt Lake Valley and Utah Valley. Carling et al. (2020), based on a two‐member mixing model, suggests that most of the dust along the Wasatch Front, including sites close to GSL playa, comes from the Sevier Dry Lake playa as opposed to the GSL playa. Back trajectory modeling for a single dust storm (Skiles et al., 2018), as well analysis of satellite data (Hahnenberger & Nicoll, 2014) suggest that southerly sources, like the Sevier Dry Lake playa, as well as Tule Dry Lake, Dugway Proving Grounds, and the Milford Flat burn areas contribute dust to Salt Lake City. Since 2019, the flux, grain size, and element data of our samples suggest that road dust, construction, and mining may play a larger role in dust exposure in cities along the Wasatch Front than previously thought. However, the GSL has shrunk since the time of these studies and has sustained low lake levels. The protective crust on the lakebed has been further weathered, potentially allowing for increased contributions of playa dust to the Northern and Central Wasatch. As long as lake levels are low, playa weathering will continue, suggesting that the playa will only become more emissive with sustained low lake levels.

#### 4.1.3 Strontium Isotope Signature of Lake Bonneville Paleo‐Shorelines

Our strontium isotope and grain size analysis data support the possibility that a large proportion of the geogenic dust deposited in urban areas may originate from disturbed paleo Lake Bonneville sediments including city soils and material mined at local gravel pits. Specifically, the lower isotope ratios observed in the vesicular soil horizon samples and GSL proximal dust collection sites relative to dust and surface sediment samples from the playa, suggest that dust from paleo‐Lake Bonneville sediments could exhibit low strontium isotope ratios. By proximity, both of these sites are more likely to receive GSL playa contributions than urban sites, which explains why they indicate higher isotope ratios than many southerly urban sites. However, both sites indicate much lower strontium isotope ratios than expected from their proximity to the playa and instead more closely match samples from North Salt Lake, Bountiful, Ogden, and downtown Salt Lake City. Although it is likely, based on satellite imagery, that Sevier Dry Lake playa sediments (e.g., Carling et al., 2020) or dust from other regional sources contributes to the dust deposited here, we suggest it is plausible that these sites simply receive a mixture of dust from locally disturbed paleo‐lakeshore sediments, regional playas including the Sevier Dry Lake playa, as well as the GSL playa. This is supported by our grain size data which suggests the importance of contributions of local sediments to city dust, and contrasts with the prior assumption that low strontium isotope ratio dust measured along the Wasatch Front comes exclusively from the Sevier Dry Lake (e.g., Carling et al., 2020).

### 4.2 Specific Anthropogenic Activities Contribute Trace Metals in Particulate Matter

Although the trace element pollutants we discuss (Table 2) occur naturally in parent rocks and metallic minerals, they are also introduced to the environment through human activities, such as fossil fuel combustion, mining and smelting, metallurgy, waste incineration, transportation, and agriculture (Adriano, 2001). Through contributions of nearby metal‐rich playas (Perry et al., 2019), and historical and current metal‐emitting industrial activities, Northern Utah has multiple sources contributing metals‐laden dust and particulate pollution to our samples. We interpret enrichment relative to upper continental crust and groupings of trace elements in our data set using principal component analyses to separate elements that may be more likely to originate from anthropogenic particulate pollution from those that are likely to originate from the GSL playa. Because the metals concentration in the playa sediments is heterogeneous, some elements may be traced to both direct deposition of pollution as well as transport of contaminated playa sediments.

Our PCA produces similar elemental groupings to those observed in Goodman et al. (2019), but unlike their PCA results, our principal components do not explain a large amount of the variance in the geochemistry data set (<20%). The low variance explained in our data set arises because anthropogenic trace elements come from different sources and do not covary over our spatial domain. While we cannot address the possible sources of all metals measured in this data set, we can address likely sources of specific metals on the EPA Priority Pollutants list that have large or persistent residential RSL exceedances in our dust data set.

#### 4.2.1 Lanthanum

The spatial structure in our data set suggests that most La comes from oil refineries, with possible additions of La transported from dust on the GSL playa. Within the Perry et al. (2019) surface sediment data set, the highest relative abundances of La were detected in the northern part of the Bear River Bay (30–40 mg kg^−1^), co‐varying with other REEs, Tl and Al. If the majority of the La in our dust samples came from Bear River Bay dust sources, we would expect our dust trap data would (a) indicate strong covariance between the relative abundances of La, Al and other REEs (b) indicate higher La and other REE relative abundances at sites closer to the Bear River Bay hotspots, like the Ogden (O2) and Farmington Bay sites (NEF and SWF). Instead, we observe the highest relative abundances of La, but not other REEs or Al, in the dust traps closest to the oil refineries. This is consistent with the assessment by Goodman et al. (2019), who suggested that the high La abundance and high La/Ce ratios could be traced to emissions from oil refining.

#### 4.2.2 Thallium, Copper, and Molybdenum

General sources of Tl to the environment include mining and smelting, metallurgy, fossil fuel combustion, electronics industry, cement industry, and waste incineration (Table 2). In our dust data set, the strong correlations with Cu (Figure S6 in Supporting Information S1) and Mo, two abundant metals present in the Bingham Canyon mine deposit, suggest that much of the observed Tl in Salt Lake Valley dust traps can be attributed to direct particulate pollution from copper mining, concentrating and/or smelting activities. With our data set, it is difficult to determine whether processing activities like smelting and concentrating contribute more Cu and Tl to dust in the city, relative to direct dust transport from the open pit mine or tailings piles. Other possible sources of Tl to dust include transport of GSL playa sediments which contain high relative abundances of Tl, particularly in northern Bear River Bay (Perry et al., 2019).

#### 4.2.3 Arsenic

Because As and V in dust indicate a moderately strong correlation, and tend to be present in lower relative abundances at northern sites near GSL playa and in higher relative abundances at southerly sites far from GSL playa, we infer that the sources of As and V to urban dust deposition are linked and unlikely to arise from playa dust. Both trace elements may be traced to early 1900s herbicide and pesticide application to orchards and crops (Church, 2004; Knowlton et al., 1947) or 1950s–1970s suburban lawn herbicide application for species like crabgrass (Folkes et al., 2001), as well as coal combustion (e.g., home heating). Because both tend to occur in higher relative abundances in urban and exurban wealthier neighborhoods (SHP, HW, H3, and LEHI), we suggest that they may follow locations of historical agricultural land, or neighborhoods that were historically wealthy enough to heat their homes with coal. Other possible sources of these trace elements include Kennecott mine tailings piles (Miller, 2020; Perry et al., 2019) or direct releases from mining operations (US Environmental Protection Agency, 2021), and tailings from lead‐arsenic milling and concentrating that occurred in the Salt Lake Valley in the early twentieth century (Church, 2004; Knowlton et al., 1947). Arsenic patterns in lake sediments of a Uintas lake also capture Lead‐Arsenic smelting activities in the Salt Lake Valley (Reynolds et al., 2010).

We do not have the sample spatial or temporal granularity to tease apart the contributions of each of these numerous possible sources. However, assuming that one or more within‐city sources of As contribute to dust in the Salt Lake Valley is consistent with a few lines of evidence. First, we have demonstrated that urban dust samples and playa dust samples are geochemically distinct (Figures 2, 3, 4). Second, our data suggest that local mining, construction and road dust contribute to dust loads within the city. Third, prior studies have reported spatial variability of As in groundwater, including high levels in the northwestern part of the Salt Lake Valley groundwater basin (e.g., Figure 3; Miller, 2020). While this finding of diffuse and local As sources likely contributing to As in urban particulate matter does not negate the importance of As contributed by GSL playa dust emissions to urban areas, we do suggest that risk assessments for dust‐mediated As ingestion also include consideration for the various additional sources of As to particulate matter deposition in the Salt Lake Valley.

### 4.3 Implications of Trace Metals Exposure From Dust Deposition for Marginalized Groups in the Salt Lake Valley

Lead (Pb), Tl, and Ni tended to be higher in dust collected at the ROSE and UDAQ Air Monitoring Center (AMC) exposure sites (Figure 2). These two sites are situated west of the I‐15 industrial and commercial corridor in neighborhoods composed of 40%–50% residents of Latino ethnicity and 38%–44% white residents (United States Census Bureau, 2020). ROSE was classified by the Home Owners Loan Corporation as “hazardous” in 1940, while the neighborhood for the AMC was not rated but is within a mile of a neighborhood that was rated “hazardous” (Nelson et al., 2021). Among our sampling sites, these are closest to an area with the highest density of permitted emitters present in the Salt Lake Valley (U.S. Environmental Protection Agency, 2022). In contrast, the Hawthorne Elementary (HW) and Sugarhouse Park (SHP) sites, situated east of the I‐15 industrial and commercial corridor, were “still desirable” and “definitely declining” (due to the siting of the now‐demolished Sugar House Prison) respectively on the zoning map (Nelson et al., 2021). These sites are in neighborhoods with more than 80% white residents. All of these sites are within 10 miles of one another near the Salt Lake City downtown.

Our findings do not unequivocally suggest that non‐white and lower income communities are exposed to more hazardous metals in their dust, especially as we found that high income very white areas received more As in their dust than lower income areas. However, prior studies within the Salt Lake Valley and in other major cities found evidence of higher pollution exposures for non‐white residents. Likewise, EPA permitted emitters tend to be clustered in neighborhoods with higher proportions of non‐white people in the study area (U.S. Environmental Protection Agency, 2022). Cobley et al. (2021) found that foliar chemistry in lower income, less white, higher traffic neighborhoods in the Salt Lake Valley indicated higher levels of ozone and NO_*x*_ air pollution. In Demetillo et al. (2021), Salt Lake City was identified (among other major cities) as having disproportionate exposure of Hispanic, Black, Asian, Native American, and low‐income residents to NO_2_ air pollution. This disproportionate effect is most evident during good air quality days, and attenuates during long‐duration persistent cold air pool pollution events (Mullen et al., 2020), where all residents experience very poor air quality. However, even among air pollution events, lower income, less white students are more likely to miss school (Mendoza et al., 2020). These findings are not unique to the Salt Lake Valley, and instead follow patterns of environmental injustice present in municipalities across the United States (Castillo et al., 2021; Demetillo et al., 2021).

Because of our sampling strategy and limitations on our understanding of exposure and health implications of metals in dust, we cannot draw conclusions about whether dust exposure in any neighborhood arises due to high intensity short duration events (e.g., dust storms) or chronic exposure (e.g., small doses of daily dust from local sources).

## 5 Conclusions

We quantified depositional dust fluxes and trace metals delivery to populated areas along Utah's Wasatch Front, and compared their relative abundances to those we might expect if dust arrived unaltered from the GSL playa. This work is one of few published that specifically evaluates delivery of trace‐element enriched particulate matter to urban areas and utilizes spatial structure to evaluate sources of metal and trace element pollution.

We found that the built environments of cities decrease depositional dust fluxes, especially decreasing deposition of larger grain sizes. However, exurban communities remain vulnerable to dust exposure from GSL and other playas. We suggest that the majority of dust deposited to our exposure‐level samplers in urban areas is generated from local soil material, with smaller contributions from regional playas. Local industries and soil contamination contribute trace metal particulate pollution to exposure‐level and higher elevation samplers, yielding dust i.e., enriched in trace metals associated with industrial, mining, and other anthropogenic activities. Our results do not preclude increases in GSL playa dust fluxes that may be likely to occur with sustained low lake levels and physical and chemical weathering of the protective salt crust that covers much of the current extent of the playa.

Evaluating the risk of exposure to dust‐associated metals is challenging because no standards have been developed. However, we contextualized our results using EPA's soil regional screening levels. We found high levels of metals including La, Cu, Tl, Mo, As, among others that exceeded RSLs at our collection sites. These metals could be linked to particulate pollution from local industries including oil refineries, copper mining, concentrating and processing as well as legacy activities like fertilizer and pesticide application and coal burning. We suggest that some of these constituent patterns may also be present in PM_2.5_, and suggest additional work to constrain that possible risk. We caution that depositional dust sampling, as performed in this study, does not fully represent the human health risks that arise from (a) high‐intensity short duration events like dust storms or (b) the airborne (pre‐deposition) dust inhalation exposure rates experienced by residents.

In summary, long‐term vulnerability to dust and particulate pollution air quality issues may be sensitive to within‐city current and historical industrial, agricultural and land use practices in addition to GSL dessication and playa weathering and contributions of regional dust sources. We emphasize that dust fluxes from the GSL playa are likely to be non‐linear and thus risks to the local populace are also likely to be non‐linear. If the lake remains at a sustained low level allowing the protective salt crust to be physically and chemically weathered, a greater proportion of the playa is likely to become emissive and existing emission locations may become more emissive. This may be particularly problematic for residents living in the northern extent of the Salt Lake Valley and residents of Davis, Weber, Box Elder and Cache counties.

## Conflict of Interest

The authors declare no conflicts of interest relevant to this study.

## Supporting information

Supporting Information S1

## Data Availability

Data are publicly available on ScienceBase as a US Geological Survey Data release (Blakowski et al., 2022 ).
