# Supplementary material for: Industrial Particulate Pollution and Historical Land Use Contribute Metals of Concern to Dust Deposited in Neighborhoods Along the Wasatch Front, UT, USA
Source: Geohealth. 2022 Nov 1;6(11):e2022GH000671. doi: 10.1029/2022GH000671 (PMC9627553; doi:10.1029/2022GH000671)
Supplement: Supplementary file 1 — Supporting Information S1 [file GH2-6-e2022GH000671-s001.docx]

Supplemental Information for:

Industrial particulate pollution and historical land use contributes metals of concern to dust deposited in neighborhoods along the Wasatch Front, UT, USA

Annie Putman et al.

Table of contents:

**Text:**

Text S1: Considerations for comparing dust to Soil RSLs

**Figures:**

Figure S1: Comparison of results from duplicate analyses for 26 samples.

Figure S2: Spatial distribution of dust flux from fine grained sediments (<63 μm)

Figure S3: Spatial distribution of percentage of total dust flux from fine grained sediments (<63 μm)

Figure S4: Trace element enrichment relative to upper continental crust by group

Figure S5: Trace elements where at least one sample exceeds the EPA residential RSL for soil

Figure S6: Arsenic distributions in our samples by sampler location.

**Tables:**

Table S1: Sampler deployment and collection dates

Table S2: Explaining variations in the arsenic on the playa at in city dust traps

Text

**Text S1:** Because no RSLs thresholds have been developed for dust, we used RSLs developed for soil, which correspond to a cancer target risk of 1 in 1 million and a target hazard quotient of 0.1 for adults, to evaluate the potential for dust to adversely impact human health. Generic soil RSLs, as used in this study, were developed to assess the targets for remediation of soil at EPA superfund sites. RSLs assume total soil exposure, and do not consider trace element bioavailability or particle size within particular soils. The calculations include estimates of soil dispersion by wind and allow only for ingestion uptake pathways over long time periods (United States Environmental Protection Agency, 1991). This means that RSLs may not represent a complete exposure threshold for dust, as the inhalation pathway and transference of a metal from lung tissue to blood is not evaluated.

Due to differences in total soil ingestion and body size, RSLs are lower for children, and higher for adults. We present our data relative to generic RSLs. Because dust events occur episodically, while chronic soil exposure occurs almost daily for residents, and soils may have additional geogenic material diluting metals, using an EPA RSL to evaluate our data may be conservative. However, because our data represent the maximum possible bioavailable fraction due to our leach method, whereas the calculations are performed for total soil, where some proportion of the sample may not be bioavailable, using EPA RSLs may not fully characterize the risk to individuals, and especially children. Likewise, this method for assessing exposure risk does not consider the health effects of exposure to multiple contaminants at the same time.

Figures


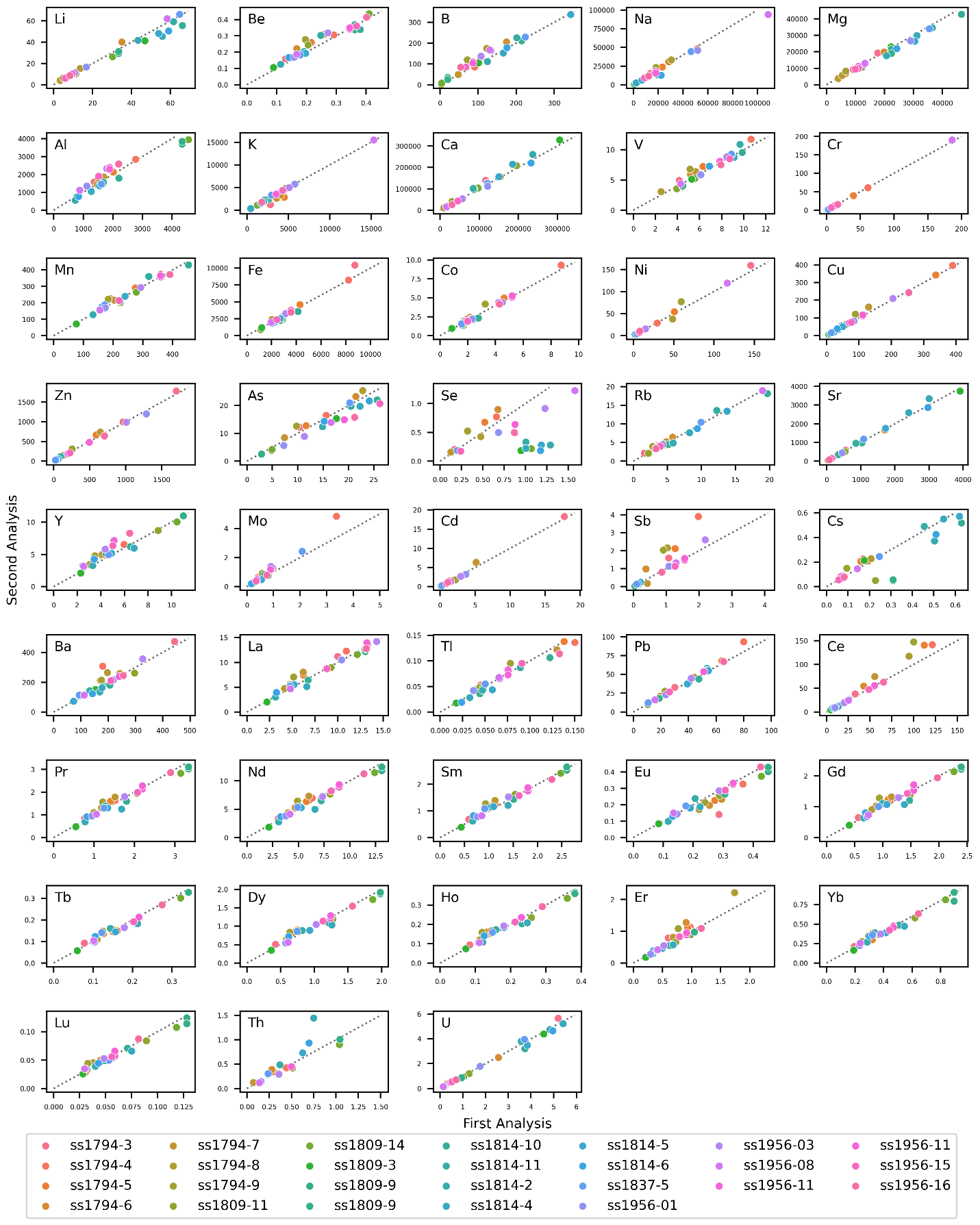


Figure S1: Comparison of results from duplicate analyses for 26 samples. Dotted line indicates a 1 to 1 relationship.


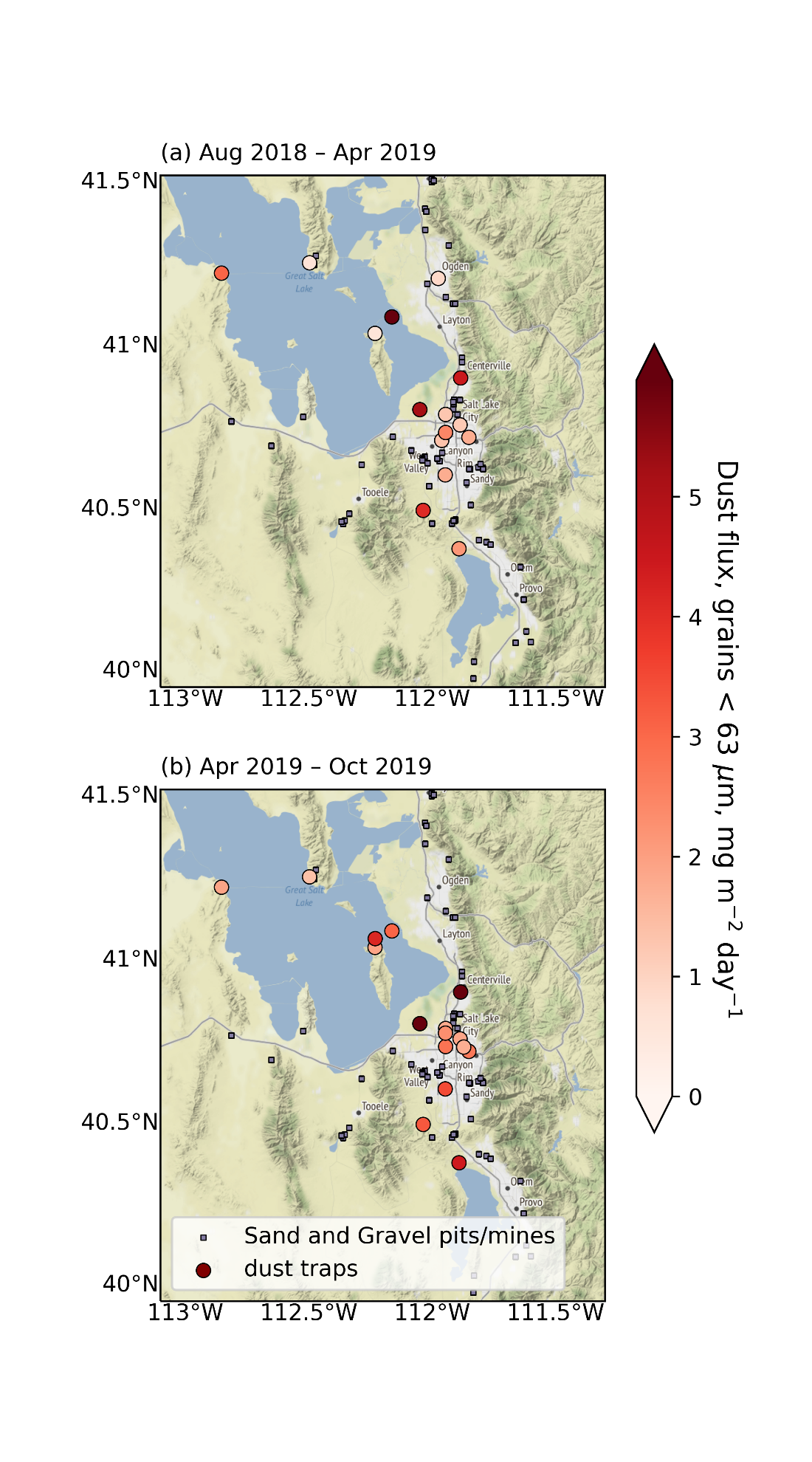


Figure S2: Spatial distribution of dust flux for grains smaller than 63 μm for both collections


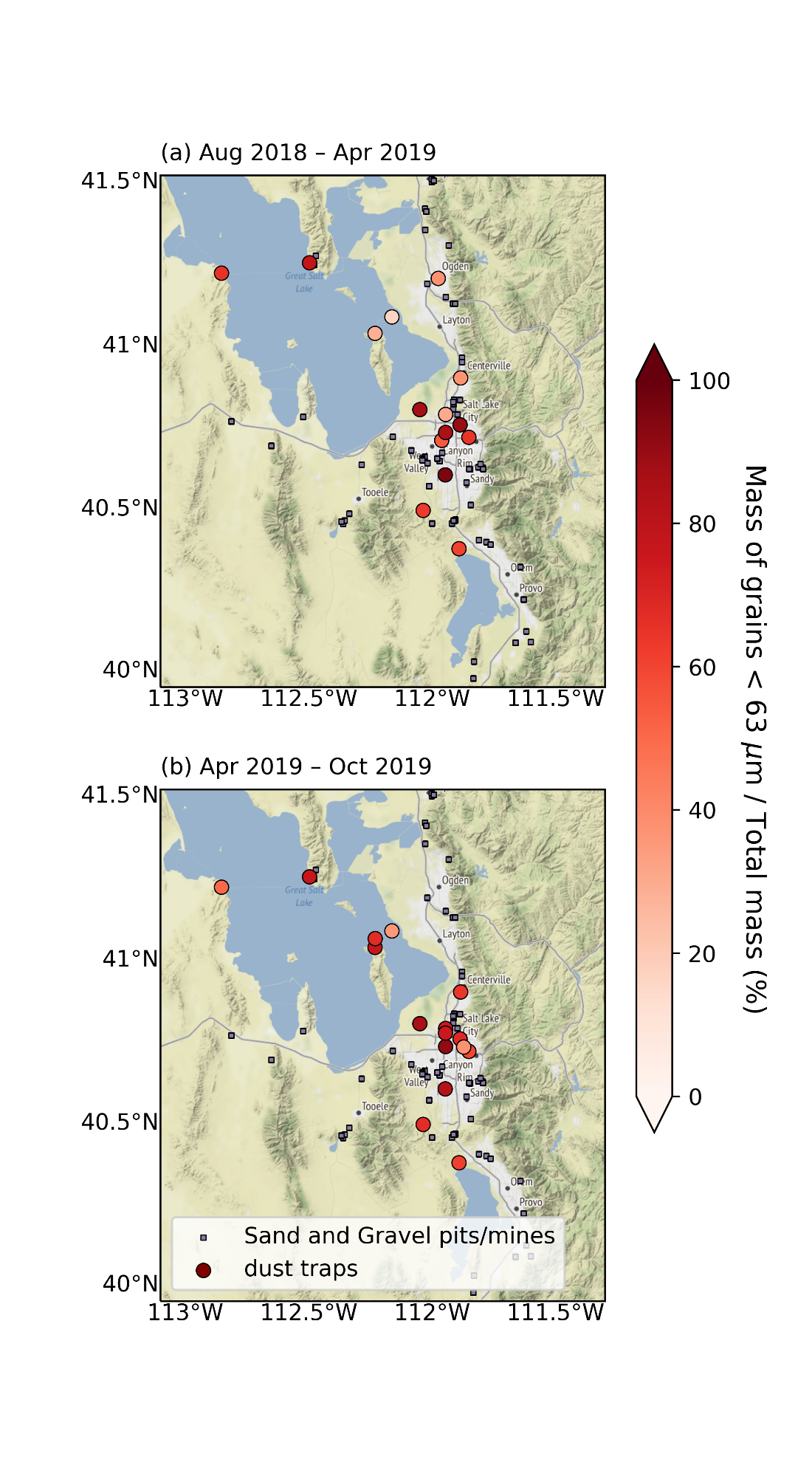


Figure S3: Spatial distribution of percentage of total dust flux from grains smaller than 63 μ for both collections..


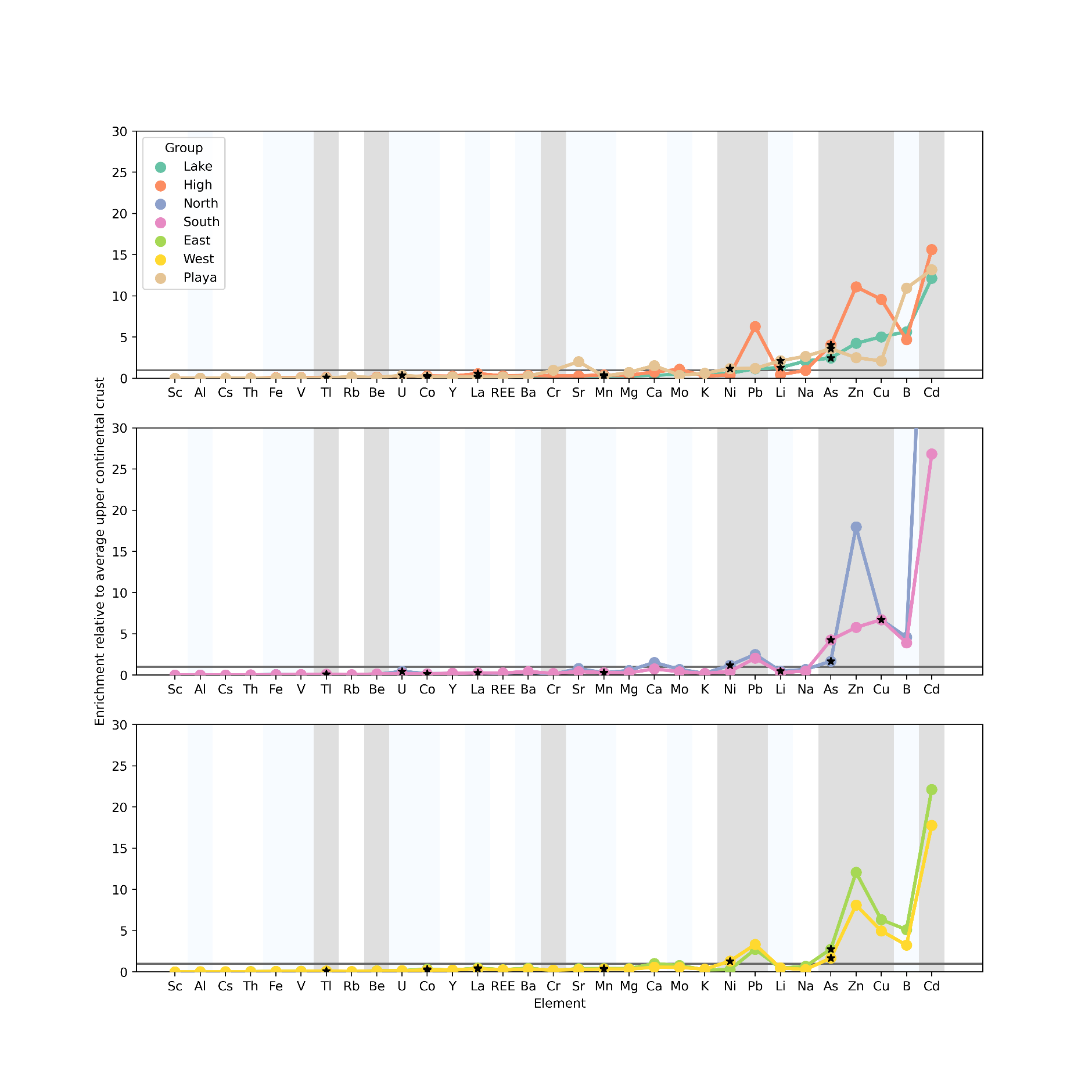


Figure S4: Enrichment of mass-weighted average trace element relative abundances relative to upper continental crust. Starred elements are those where at least one collection in the group composing that value indicated exceedance of the EPA RSL for that element. Elements are ordered by the enrichment of the lake samples.


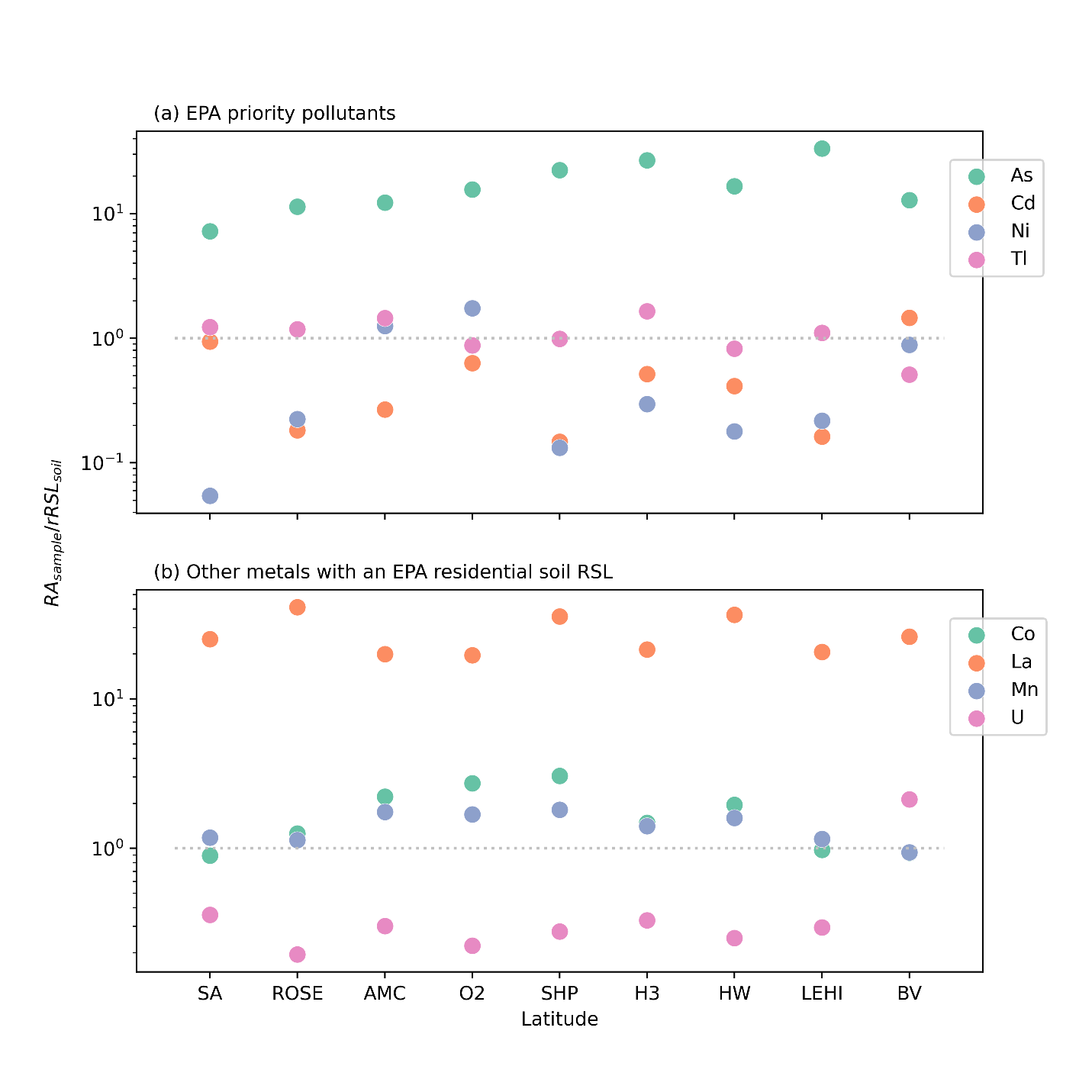


Figure S5: Trace elements where at least one flux weighted sample exceeds the EPA residential RSL for soil (rRSL_soil_). Data are categorized by site and the ratio of the relative abundance of the trace element in the sample to the residential RSL value is plotted on the y axis. Values less than 1 indicate that the relative abundance of the trace element in pollution is less than the EPA soil RSL. Values of 1 or greater indicate that the dust sample exceeds to EPA soil RSL. Note that we are comparing dust to a soil metric. It is likely that in soil, these metals abundances would be diluted by other soil materials. However, the RSLs allow us to determine which elements in dust may be cause for the greatest concern.


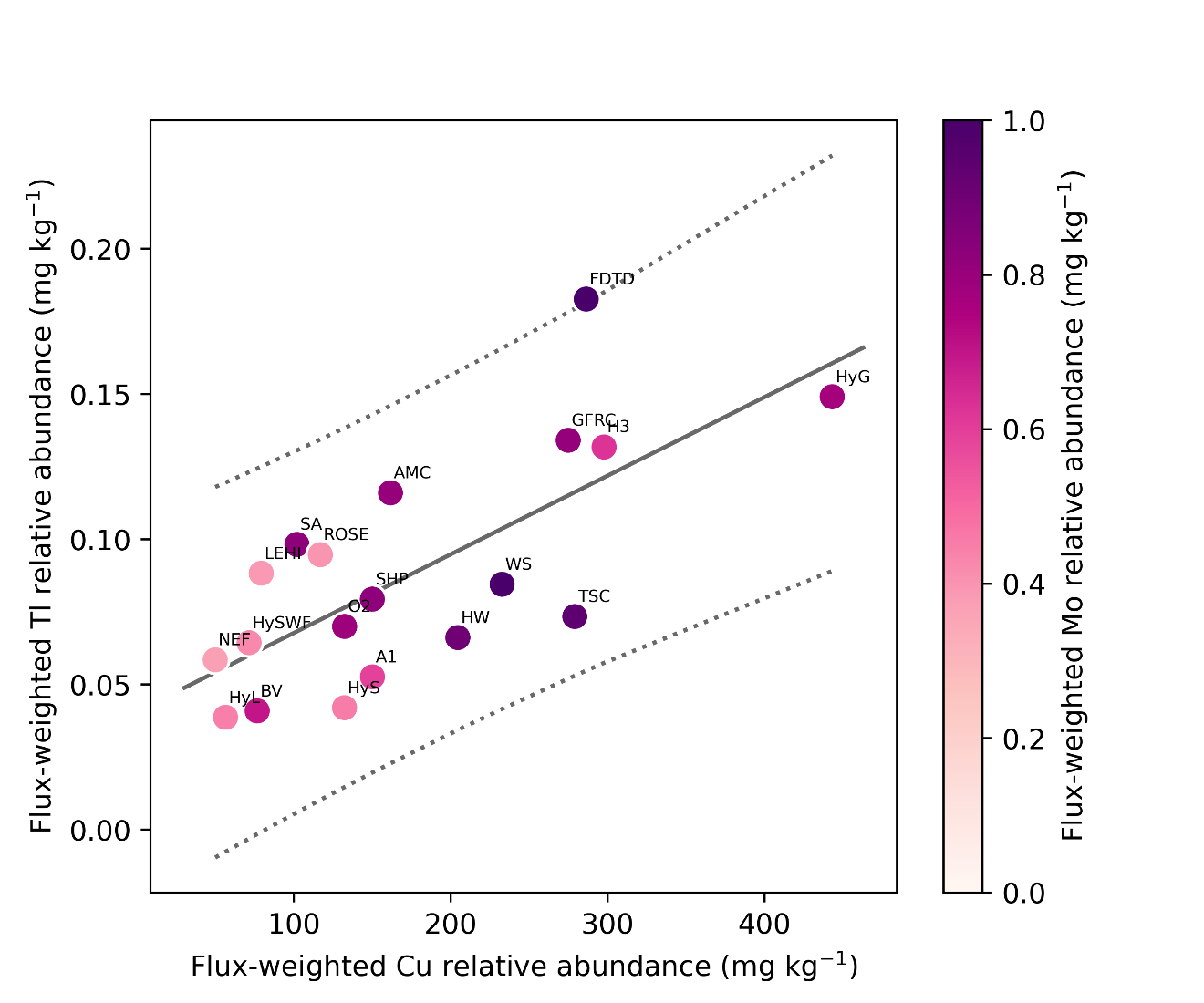


Figure S6: Copper and molybdenum flux-weighted averages predict thallium flux weighted average values across all dust traps.

Tables:

*Table S1: Sample deployment and collection dates*

| Site ID | Start Date 1 (pilot) | Collection Date 1 (pilot) | Start Date 2 | Collection Date 2 | Start Date 3 | Collection Date 3 |
| --- | --- | --- | --- | --- | --- | --- |
| A1 |  |  | 7/26/2018 | 4/25/2019 | 4/25/2019 | 10/24/2019 |
| AMC |  |  | 7/26/2018 | 4/24/2019 |  |  |
| BV |  |  | 7/26/2018 | 4/24/2019 | 4/24/2019 | 10/24/2019 |
| O2 |  |  | 7/26/2018 | 4/24/2019 |  |  |
| SA |  |  | 7/26/2018 | 4/22/2019 | 4/22/2019 | 10/24/2019 |
| HyL | 3/8/2018 | 8/2/2018 | 8/2/2018 | 4/23/2019 | 4/23/2019 | 10/28/2019 |
| HyS | 3/7/2018 | 8/2/2018 | 8/2/2018 | 4/23/2019 | 4/23/2019 | 10/28/2019 |
| H3 |  |  | 8/10/2018 | 4/24/2019 | 4/24/2019 | 10/24/2019 |
| NEF |  |  | 8/10/2018 | 4/25/2019 | 4/25/2019 | 10/24/2019 |
| WS |  |  | 10/29/2018 | 4/22/2019 | 4/22/2019 | 10/23/2019 |
| FDTD |  |  | 11/5/2018 | 4/24/2019 | 4/24/2019 | 10/23/2019 |
| GFRC |  |  | 11/20/2018 | 4/22/2019 | 4/22/2019 | 10/23/2019 |
| SHP |  |  | 11/20/2018 | 4/22/2019 | 4/22/2019 | 10/23/2019 |
| LEHI |  |  | 12/20/2018 | 4/24/2019 | 4/24/2019 | 10/29/2019 |
| ROSE |  |  | 12/20/2018 | 4/24/2019 | 4/24/2019 | 10/29/2019 |
| HW |  |  |  |  | 4/24/2019 | 10/24/2019 |
| HyG | 3/6/2018 | 8/2/2018 |  |  |  |  |
| HySWF | 3/6/2018 | 8/2/2018 |  |  | 4/25/2019 | 10/24/2019 |
| TSC |  |  |  |  | 4/24/2019 | 10/24/2019 |
| AV1 |  |  |  | 4/23/2019 |  |  |
| AV2 |  |  |  | 4/23/2019 |  |  |
| HyLD |  |  |  | 4/23/2019 |  |  |
| RVI |  |  |  | 4/25/2019 |  |  |
| FN.S |  |  |  | 4/25/2019 |  |  |
| FS.S |  |  |  | 4/25/2019 |  |  |
| FWMA.S |  |  |  | 4/25/2019 |  |  |
| HyLP.S |  |  |  | 4/23/2019 |  |  |
| HySB.S |  |  |  | 4/23/2019 |  |  |
| HySP.S |  |  |  | 4/23/2019 |  |  |
| NP.S |  |  |  | 4/23/2019 |  |  |
| RDF.S |  |  |  | 4/25/2019 |  |  |
| RP1.S |  |  |  | 4/25/2019 |  |  |
| RP2.S |  |  |  | 4/25/2019 |  |  |

Table S2: comparison of regression results explaining As variation. For surface sediments on the Great Salt Lake, As relative abundance largely tracks strontium, and is well explained by a linear combination of the relative abundances of three characteristic playa trace elements (this regression performs better than Sr alone). However, when the same regression is applied to urban dust trap samples, there is no statistical relationship between the relative abundances of characteristic playa elements and As relative abundance.

|  | Sr | Mg | Li | Intercept | Rsq | pval |
| --- | --- | --- | --- | --- | --- | --- |
| Perry et al., 2019 | **0.0084**  **( +\- 0.0016 )** | **0.0004**  **( +\- 0.0001 )** | **-0.07**  **( +\- 0.02)** | **10.16**  **( +\- 4.56 )** | 0.39 | <0.001 |
| Urban samples from this study | 0.0186  ( +\- 0.0289 ) | -0.001  ( +\- 0.0009 ) | 1.05  ( +\- 0.75 ) | **14.29**  **( +\- 5.92 )** | 0.15 | 0.55 |

References

United States Environmental Protection Agency. (1991). Risk Assessment Guidance for Superfund (RAGS). In (p. 19-31). Retrieved from https://www.epa.gov/1172
